# Vaccination against tumour endothelial marker Robo4 inhibits tumour growth

**DOI:** 10.1093/immadv/ltag005

**Published:** 2026-06-25

**Authors:** Fernanda Escobar-Riquelme, Muhammet Ali Kara, Michael J Price, Angela Hidalgo-Gajardo, Hayley L Carr, David Bending, Roy Bicknell, Natalia Savelyeva, Kai-Michael Toellner, Yang Zhang

**Affiliations:** Department of Immunology and Immunotherapy, School of Infection, Inflammation and Immunology, College of Medicine and Health, University of Birmingham, Birmingham, United Kingdom; Department of Immunology and Immunotherapy, School of Infection, Inflammation and Immunology, College of Medicine and Health, University of Birmingham, Birmingham, United Kingdom; Immunology Program, The Babraham Institute, Babraham Research Campus, Cambridge CB22 3AT, United Kingdom; Department of Immunology and Immunotherapy, School of Infection, Inflammation and Immunology, College of Medicine and Health, University of Birmingham, Birmingham, United Kingdom; Department of Immunology and Immunotherapy, School of Infection, Inflammation and Immunology, College of Medicine and Health, University of Birmingham, Birmingham, United Kingdom; Departamento de Fisiopatología, Facultad de Ciencias Biológicas, Universidad de Concepción, Concepción, Chile; Bioinformatics, the Babraham Institute, Babraham Research Campus, Cambridge, United Kingdom; Department of Immunology and Immunotherapy, School of Infection, Inflammation and Immunology, College of Medicine and Health, University of Birmingham, Birmingham, United Kingdom; Department of Cardiovascular Sciences, College of Medicine and Health, The University of Birmingham, Birmingham, United Kingdom; Molecular and Clinical Cancer Medicine, Institute of Systems, Molecular and Integrative Biology, Faculty of Health and Life Science, University of Liverpool, Liverpool, United Kingdom; Department of Immunology and Immunotherapy, School of Infection, Inflammation and Immunology, College of Medicine and Health, University of Birmingham, Birmingham, United Kingdom; Immunology Program, The Babraham Institute, Babraham Research Campus, Cambridge CB22 3AT, United Kingdom; Department of Immunology and Immunotherapy, School of Infection, Inflammation and Immunology, College of Medicine and Health, University of Birmingham, Birmingham, United Kingdom; Immunology Program, The Babraham Institute, Babraham Research Campus, Cambridge CB22 3AT, United Kingdom

**Keywords:** immunotherapy, conjugate vaccines, angiogenesis, cancer, B cells, antibody

## Abstract

**Introduction:**

Targeting tumour antigens is a major challenge in cancer-immunotherapy. We use active vaccination to induce antibodies targeting self-antigen Robo4, which is selectively expressed on tumour vascular endothelium, and supports vascular development. Our previous work showed that a conjugate of Robo4 with a foreign carrier protein induced autoantibodies specific to Robo4, which inhibited angiogenesis and tumour growth.

**Methods:**

To translate the vaccine protocol to exploit a carrier protein used in routine human vaccination schedules, the well-characterized, non-toxic fragment C of tetanus toxin (TTc) was selected as the carrier protein. Recombinant protein Robo4-TTc (R4-TTc) was produced by Robo4 genetically linked to TTc.

**Results:**

Priming with the carrier TTc followed by boost with Robo4-TTc (R4-TTc) efficiently induces strong antibody responses to Robo4 and inhibits tumour growth in LLC1 and 4T1 tumour models. The growth inhibition was correlated with anti-Robo4 IgG1 titres. Furthermore, decreased vessel formation and increased immune cell infiltration in tumours from R4-TTc vaccinated mice in the absence of detectable adverse effects on health.

**Conclusion:**

The data indicate that this vaccination strategy remodels tumour vessels and probably promotes immunogenic pathway activation, therefore repressing tumour growth.

## Graphical Abstract

**Graphical Abstract:**
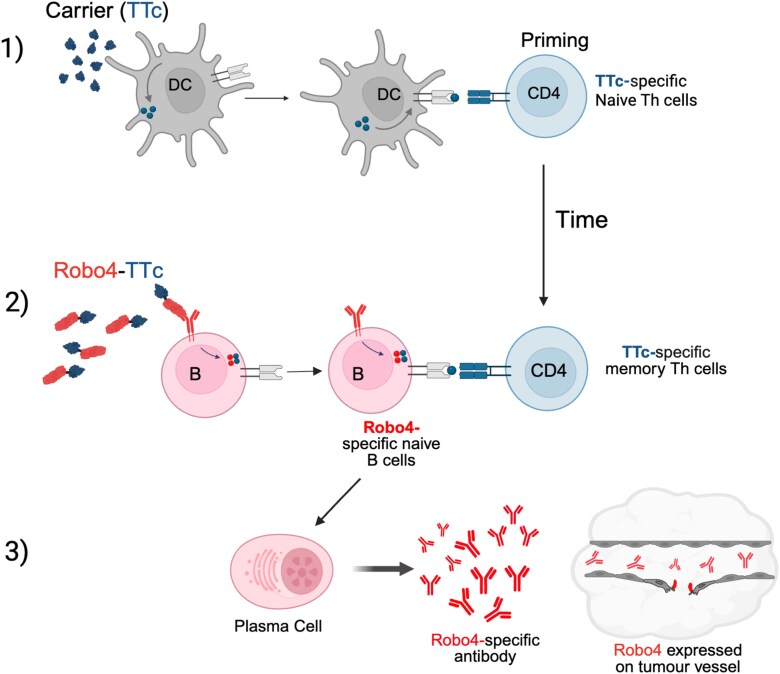

## Introduction

Tumour growth critically depends on the supply of nutrients through tumour associated vasculature [1]. Tumour associated vasculature has an abnormal morphology, showing reduced blood perfusion and shear stress, which limits drug access and immune response in solid cancers. Low shear stress at the endothelial surface in tumour vessels induces the expression of specific proteins, called tumour endothelial markers (TEMs). Targeting TEMs can impede tumour growth in animal models [1], because localization at the endothelial surface grants accessibility to the immune system (e.g. immune cells and antibodies). Remodelling of vessel shape and function leads to blood clots, thereby destroying the surrounding tumour tissue, and inducing immune cell infiltration [2, 3]. Finally, tumour vascular endothelial cells are more genetically stable than the malignant cells that produce novel and mutant proteins (called neoantigens), which reduces the likelihood of the emergence of mutants [3].

Robo4 (or Magic Roundabout) is a transmembrane protein, and the fourth member of the roundabout (Robo) family [4]. It is involved in angiogenic sprouting, filopodia formation and maintenance of the endothelial barrier [5]. Robo4 is selectively expressed in vessels of tumour tissues including pancreatic, breast, lung, and prostate cancers, and to a lesser extent on healthy mature vasculature, identifying it as a potential target for suppressing pathological angiogenesis [6–9].

Monoclonal antibodies are used in cancer immunotherapy. Passively administered antibodies are expensive to produce and store, and have limited retention in patients. Active vaccination is a common method to induce antibody production and cytotoxic T-cell mediated immunity [10]. It is cost-efficient, can target multiple epitopes, and generate a long-lasting response. However, triggering an antibody response to self-antigen can be difficult because of central immune tolerance, which limits the availability of antigen-specific CD4^+^ T helper cells that support B cell activation to the autoantigen [11]. We previously developed a conjugate vaccine that efficiently induces B cell activation to the self-antigen Robo4 by chemically conjugating Robo4 to the foreign carrier protein chicken gamma globulin (CGG) (Robo4-CGG), which resulted in the production of Robo4 specific antibodies in CGG primed mice and moderated tumour suppression in the Lewis Lung Carcinoma (LLC1) model [7].

To translate this vaccine protocol for human vaccination, we chose the non-toxic fragment C of tetanus toxin (TTc) from *Clostridium tetani*, used in clinical trials [12], as a carrier protein. TTc contains a promiscuous universal MHC class II epitope, to support the development of humoral and cellular immune response through activation of CD4 T cells [13–15]. Activated CD4 T cells provide critical signals (e.g. CD40L, IL21) promoting activated B cells proliferation, isotype switching, and germinal centre formation, resulting in the generation of memory B cells, high affinity plasma cells that produce high affinity switched antibody. TTc fragment has been extensively studied as a neuroprotective agent for central nervous system disorders and as a carrier protein in different types of vaccines [16–18]. Furthermore, most patients have pre-existing immune response to tetanus antigens courtesy of routine vaccination. This ensures that Robo4-specific B cells will be able to recruit help from pre-existing memory CD4 T cells ensuring a rapid activation of humoral response.

Here, we genetically engineered the extracellular domain of Robo4 to TTc protein. This recombinant Robo4-TTc (R4-TTc) protein administered with adjuvant induced a strong anti-Robo4 humoral immune response in mice, and controlled tumour growth in LLC1 and 4T1 models. Further study demonstrated reduced tumour vascularization and increased T cell infiltration in tumours after R4-TTc protein vaccination. These anti-tumour effects, without observed adverse effects, indicate that this protein conjugate vaccine strategy has the potential to improve the results of cancer therapy.

## Results

### Carrier priming supports naïve B cells responding to poorly immunogenic antigen

**Figure 1:**
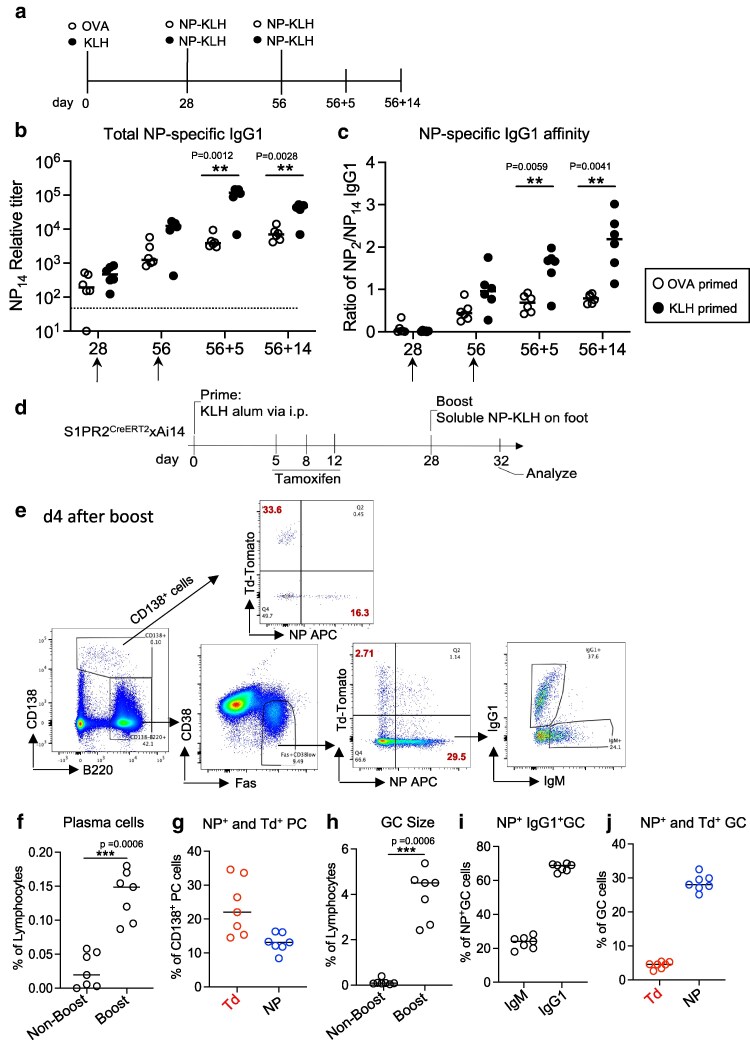
Carrier priming supports naïve B cells responding to hapten antigen. (a) Experiment protocol for B-C. C57BL6 mice were primed with carrier KLH in alum or carrier OVA in alum and then boost with soluble NP-KLH two times. (b) Total NP specific IgG1 titre. (c) NP specific IgG1 affinity. B–C: Each dot represents one animal. Two-way ANOVA with mixed-effects analysis (Tukey multiple comparisons). Dash line is the detection limit. (d) Experiment set up for E-J. S1pr2ERT2^Cre/wt^ xAi14 mice were primed with KLH in alum i.p., and then given tamoxifen at d5, 8, and 12 to track GC derived memory B cells. 4 weeks later mice were boost with soluble NP-KLH on both rear feet. Popliteal lymph nodes (LN) were analysed on day 4 after the boost. (e) FACS gating strategy. (f) Plasma cells in popliteal LN after the boost on foot. (g) NP^+^ and Td^+^ plasma cells in popliteal LN after the boost. (h) GC size in LN before and after the boost. (i) NP ^+^ IgG1^+^ GC B cells. (j) NP^+^ and Td^+^ GC cells in popliteal LN after the boost. F–I: Each dot represents one animal. Two-sided Mann–Whitney *U* test. Merged two independent experiments.

Carrier priming is an established method to boost T cell help and enhance antibody responses to poorly immunogenic antigens [10, 19]. To demonstrate the mechanism of carrier priming, a simple hapten-carrier model was selected [20, 21]. Mice were primed with carrier protein keyhole limpet haemocyanin (KLH) and boosted twice with KLH-conjugated to the small chemical hapten 4-hydroxy-3-nitrophenylacetyl (NP). Similar to self-antigens, chemical haptens alone by themselves are non-immunogenic and cannot recruit CD4^+^ T cell help [22]. Mice were primed with the unrelated antigen chicken ovalbumin (OVA) in alum served as controls (Fig. 1a). The production of hapten-specific IgG1 was analysed after immunization with soluble NP-KLH. IgG1 is the main switched antibody subclass in responses to protein antigens in alum [23]. While the first immunization with NP-KLH (d28) led to a significant increase in NP-specific IgG1 in all groups (Fig. 1b, at d56), the titres were 10× higher if mice were primed with the matching carrier KLH in alum. The second NP-KLH boost induced a further rise of NP-specific IgG1 titre and affinity in the KLH-primed group (at d56 + 5) (Fig. 1c). This confirms that the carrier-priming accelerates antibody response to a poorly immunogenic antigen.

To understand the mechanism of this carrier priming vaccination, the same model antigen NP was used to immunize S1pr2ERT2^Cre/wt^ x Ai14 mice. As S1pr2 is specifically expressed in germinal centre (GC) B cells, this allows to fate-map primary GC-derived memory B cells (mBC) in a tamoxifen-dependent manner [24, 25]. Mice were primed with carrier KLH in alum i.p., tamoxifen was administered at d5, 8 and 12 via gavage to induce the expression of TdTomato to track GC-derived KLH specific mBC in skin draining lymph nodes (Fig. 1d). Four weeks later mice were boosted with soluble NP-KLH on both rear feet. Popliteal lymph nodes were harvested 4 days after the boost. Here, TdTomato-marked (Td^+^) cells will correspond to GC-derived mBC during the carrier primary response, and NP-specificity is used to track the recruitment of naive B cells.

Four days after the boost, plasma cells (PCs) and GC B cells significantly increased in the boosted group compared with the non-boosted (Fig. 1e–h). Moreover, around 20% of the PCs from the boosted were Td^+^ (mBC-derived) while 10% were NP^+^ (Fig. 1g), indicating that mBC rapidly differentiated into plasmablasts/plasma cells upon reexposure to the same carrier KLH, whereas at this stage, few plasma cells are derived from newly activated naïve B cells. Within GC B cells, most of the cells (around 30%) were NP-binding, Td-negative, and IgG1 switched (Fig. 1i), whereas only a minor proportion (2–3%) were Td^+^ cells (Fig. 1j), which suggests that carrier priming induced KLH specific antibody restricts the GC entry of KLH specific mBC and efficiently recruits naïve B cells joining GC reaction [26, 27]. Taken together, this indicates that carrier-priming promotes naïve B cell responding to poorly immunogenic antigens and leads to the generation of high antibody titres and affinity. We therefore decided to test a carrier-priming approach with carrier TTc to induce the immune response of self-reactive B cells.

### Conjugate vaccine breaks immune tolerance to Robo4 and generates Robo4-specific IgG1 response

Robo4 as a non-mutated self-antigen, does not efficiently recruit T cell help due to central T cell tolerance [7]. To enhance the immunogenicity of a Robo4 vaccine, we designed a similar conjugate vaccine strategy as for NP, using the well characterized antigen TTc as a carrier. To generate the vaccine, the extracellular domain of mouse Robo4 (R4) was genetically linked to TTc (Supplementary Figs S1 and S2), and stably transfected into HEK293 cells to produce a recombinant R4-TTc protein. The protein was purified from the supernatant of stably transfected HEK293 cells (Supplementary Fig. S3). The generation and purification of the recombinant proteins TTc and R4-TTc are described in Materials and Methods (Supplementary Figs S2 and S3).

**Figure 2:**
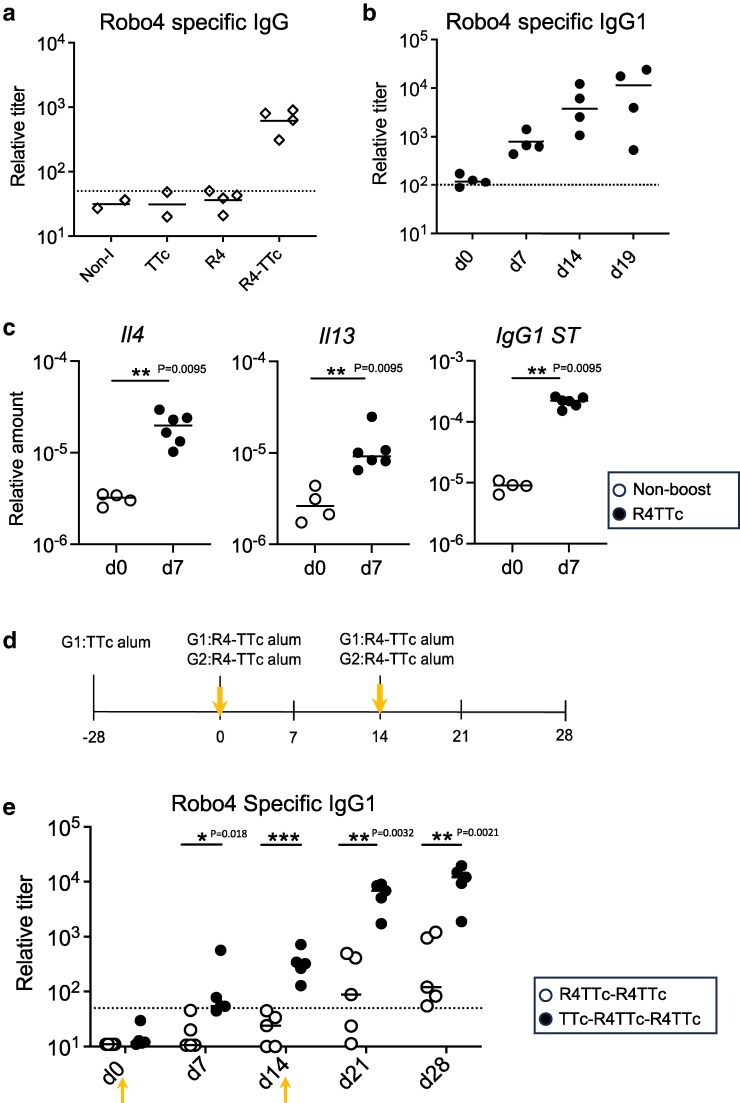
The induction of Robo4 specific antibody after injection of Robo4-TTc in Alum. (a) C57BL6 mice were i.p. immunized with purified protein TTc, Robo4 (R4), Robo4-TTc (R4-TTc) in alum, and 2 mice as non-immunized control (Non-I). Blood samples were harvested at day28. Robo4 specific IgG was measured. Dash line is the detection limit. (b) C57BL6 mice were primed with 50 µg TTc in alum and 3 weeks later injected with 40ug R4-TTc in alum. Robo4 specific IgG1 was measured. Dash line is the detection limit. (c) IL4, IL13, and IgG1 germline switch transcript (IgG1ST) expression. Gene expression on spleen section at d7 after injection of R4-TTc in alum. All values are relative to β2 m mRNA. Each dot represents one animal. Two-sided Mann–Whitney *U* test. (d) Experiment protocol. TTc in alum primed C57BL6 mice (as G1) or non-primed C57BL6 mice (as G2) were injected with R4-TTc in alum via i.p. every 2 weeks. (e) Robo4 specific IgG1 in serum. Each dot represents one animal. Dash line is the detection limit. Two-way ANOVA with mixed-effects analysis (Tukey’s multiple comparisons).

Initial experiments tested the immunogenicity of recombinant Robo4 without the TTc carrier protein (Fig. 2a). As expected, a Robo4 specific IgG antibody was observed only in R4-TTc group, compared with other control groups (TTc, Robo4 alone). The next experiment analysed responses to R4-TTc in alum after priming with carrier TTc in alum (Fig. 2b). Robo4-specific IgG1 was detectable at d7 after the immunization with R4-TTc in alum, and the antibody titre continued to increase. To screen the efficiency of T helper (Th) cell activation, gene expression of cytokines IL-4 and IL-13, and IgG1 germline transcripts as an indicator for efficient Th–B cell interaction [23, 28] were detected by qRT-PCR in spleen tissues at d7 (Fig. 2c). This showed efficient induction of CD4 T helper cells and B cell activation after the carrier-primed immunization. To explore the effect of the TTc priming strategy on the production of Robo4-specific antibody, the response between TTc-primed and non-primed animals was compared (Fig. 2d). Similar to other proteins in alum shown in Fig. 1, R4-TTc in alum mainly induces IgG1 switched antibody (Supplementary Fig. S4). TTc priming had a significant effect on the Robo4-specific antibody response (Fig. 2e), particularly R4-TTc in alum induced a detectable Robo4-specific IgG1 within 7 days. Whereas non TTc primed animals remained seronegative until the secondary immunization with R4-TTc in alum (Fig. 2e). These data suggest that conjugation with the carrier TTc efficiently enhances responses to self-antigen Robo4, probably through a combined adjuvant effect of carrier-specific antibody [29] and the recruitment of TTc-specific memory CD4 T cells, efficiently overturning B cell tolerance to Robo4.

### Robo4-TTc boost induces an efficient Robo4-specific response and retards LLC tumour growth

We have shown earlier that Robo4 vaccination can disrupt angiogenesis via antibody, particularly IgG1 mediated effects [7]. As angiogenesis promotes solid tumour growth, the effect of R4-TTc vaccination on the tumour growth from LLC1 lung carcinoma and 4T1 breast cancer models was examined.

**Figure 3:**
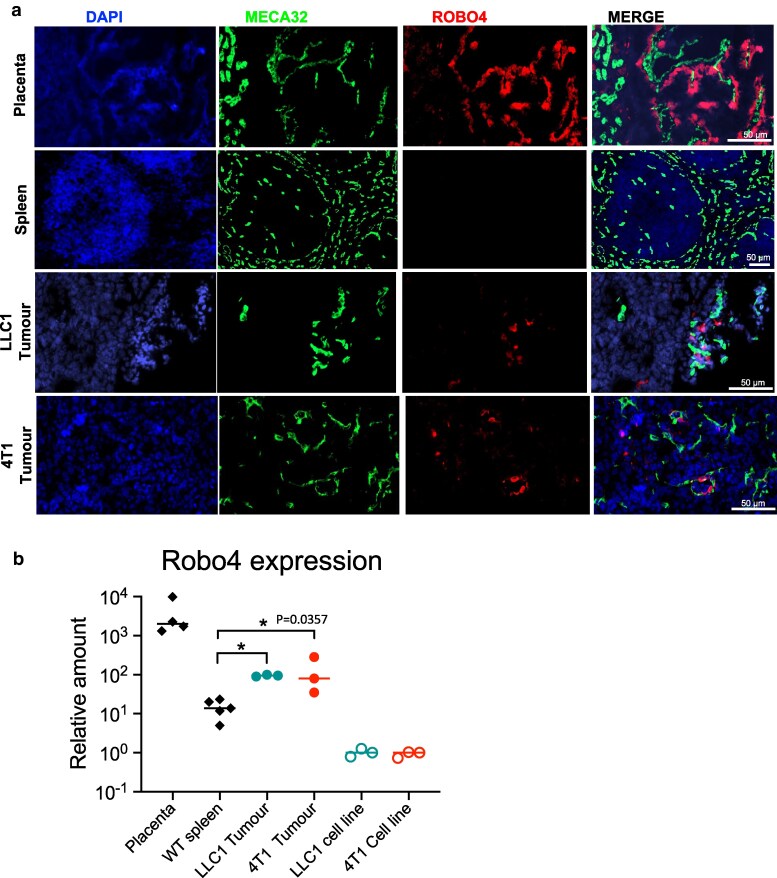
Robo4 is expressed on tumour tissue. (a) Immunofluorescent staining of the mouse mature placenta (placenta labyrinth part), adult mouse spleen, LLC1 and 4T1 tumour tissue. MECA32 stains endothelial cells. Scale bar: 50 μm. (b) Robo4 expression levels in LLC1 cell line, LLC1 tumours, 4T1 cell line, 4T1 tumours, placenta, and spleen tissue from adult wild type mice measured by qRT-PCR (β2 m as housekeeping gene). The 2^−ΔΔCT^ method was applied for relative quantification of mRNA level to tumour cell line. LLC1 and 4T1 cell lines were used as comparators. Each dot represents one animal. Two-tailed Mann–Whitney *U* test. *, Statistical significance (*P = 0.0357) is only indicated for Robo4 gene expression differences between tumour tissue and adult spleen tissue.

To show the expression of Robo4 before vaccination, endothelial cells of embryonic and adult tissues were stained with the panendothelial cell antigen (MECA32) and Robo4 antibodies (Fig. 3 and Supplementary Fig. S5). As shown by others [30], Robo4 was strongly expressed on mature placenta, particularly in the labyrinth area co-located with MECA32^+^ foetal vessels (Supplementary Fig. S5). In LLC1 and 4T1 tumours, Robo4 protein was detected in proximity to MECA32^+^ endothelia (Fig. 3a). It was not detectable in adult spleen endothelia (Fig. 3a, and Supplementary Fig. S5) in line with for Robo4 expression [31]. Further qRT-PCR analysis comparing to tumour cell line and normal spleen tissue presented that Robo4 gene expression was increased in LLC1 and 4T1 tumours (Fig. 3b). This is in line with earlier work, which screened a range of adult non-transformed tissues, and demonstrated that Robo4 expression was tumour-specific [6].

**Figure 4:**
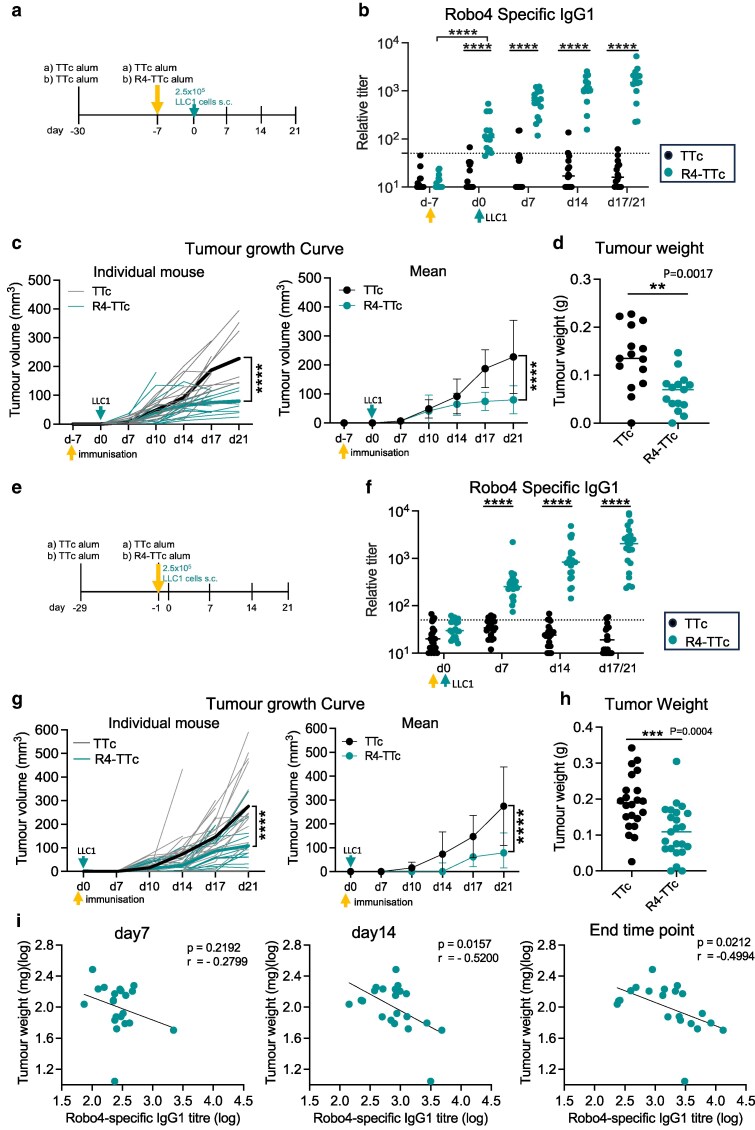
Reduced tumour growth after injection of Robo4-TTc. (a) Experiment protocol for B-D. Mice were primed with TTc in alum, 3 weeks later were boosted with R4-TTc in alum or TTc in alum. LLC1 cells were injected s.c. at d7 after the boost. (b) Robo4-specific IgG1 antibody after the Robo4-TTc in alum injection. Dash line: detection limit. Two-way ANOVA with mixed-effects analysis (Tukey multiple comparison), ****P < 0.0001. (c) Tumour growth curve. Left: Individual tumour growth curve. Bold line indicates mean. Right: Same data plotted as mean ± SD. Two-way ANOVA with mixed-effects analysis, ****P < 0.0001. (d) Tumour weight at death. Two-tailed Mann–Whitney *U* test. (b–d) Each dot represents one animal, merged two independent experiments. (e) Experiment protocol for F-I. Mice were primed with TTc alum, 4 weeks later boost with R4-TTc alum or TTc alum. LLC1 cells were injected s.c. at 1 day after the boost. (f) Robo4-specific IgG1 antibody after the Robo4-TTc injection. Dash line: detection limit. Two-way ANOVA with mixed-effects analysis (Tukey multiple comparison), ****P < 0.0001. (g) Tumour growth curve. Left: Individual tumour growth curve. Bold line indicates mean. Right: Same data plotted as mean ± SD. Two-way ANOVA with mixed-effects analysis, ****P < 0.0001. (h) Tumour weight at death. Two-tailed Mann–Whitney *U* test. (i) Correlation between the presence of Robo4-specific IgG1 at d7, d14, and end of time point, with final tumour weight. d0: when tumour cells were transplanted. Two-tailed compute Pearson correlation coefficients. (f–h) Each dot represents one animal, merged three independent experiments.

To test the effect of the vaccination protocol on tumour growth, mice were primed with TTc in alum and then challenged with LLC1 tumour cells a week after control or R4-TTc alum boost (Fig. 4a). For further tissue analysis, most tumours from both groups were harvested at a reasonable size but before reaching the severity limitation at d17 or d21. As shown before (Fig. 2e), Robo4-specific IgG1 titres were detectable within 7 days after the boost, and continued to increase until the final experiment timepoint (Fig. 4b). Tumour growth was significantly reduced in the R4-TTc vaccinated group (Fig. 4c-d). There was a negative correlation between the tumour weights and Robo4-specific IgG1 titres at d7 and d14 after tumour cell implantation (Supplementary Fig. S6B), and a negative correlation between the tumour volume and Robo4-specific IgG1 titres at d14 and the end timepoint (Supplementary Fig. S6c). These results suggest that Robo4-specific antibodies significantly inhibit LLC1 tumour growth, and that the amount of antibody produced is related to the extent of inhibition of tumour growth.

To further test the effect of anti-Robo4 antibody on different phases of tumour growth, LLC1 cells were injected s.c. within 1 day after vaccination with control or R4-TTc in alum in the TTc in alum primed mice (Fig. 4e). As described before, most tumours from both groups were harvested at the same time at d17 or 21 for further tissue analysis. In this setup no Robo4-specific antibody will be present at the time of tumour cell injection, as plasma cell generation takes at least 3 days. Only low levels of Robo4-specific IgG1 antibody were detectable by d7 after boost and continued to increase (Fig. 4f). Still, R4-TTc vaccinated mice showed significantly reduced tumour growth compared with control mice (Fig. 4g, h).

Comparing final tumour sizes with Robo4-specific IgG1 titres during the experiment showed a negative correlation at d 14 and 21 post vaccination and final tumour weight (Fig. 4i, Supplementary Fig. S7). However, there was no significant correlation with the antibody levels created at 7d post tumour transplantation. The data indicate that while Robo4 specific antibodies can reduce tumour growth, the absence of correlation between antibody titre in the first week and final tumour sizes indicates that very low to absent Robo4-specific antibody level in the first week does not negatively affect tumour growth. Antibody affects tumour growth after the first week, probably when vascularization has happened, indicating that this vaccination protocol may have therapeutic potential on established tumours.

An experiment where vaccination was done even later (7 days post tumour implantation) showed no significant tumour growth inhibition (Supplementary Fig. S8). This is not surprising, as in this setup significant antibody production only happens after d 14 (Supplementary Fig. S8b) when tumour volumes are already close to the humane endpoint.

### Robo4-TTc vaccination disrupted tumour vessel and increased immune cell infiltration

**Figure 5:**
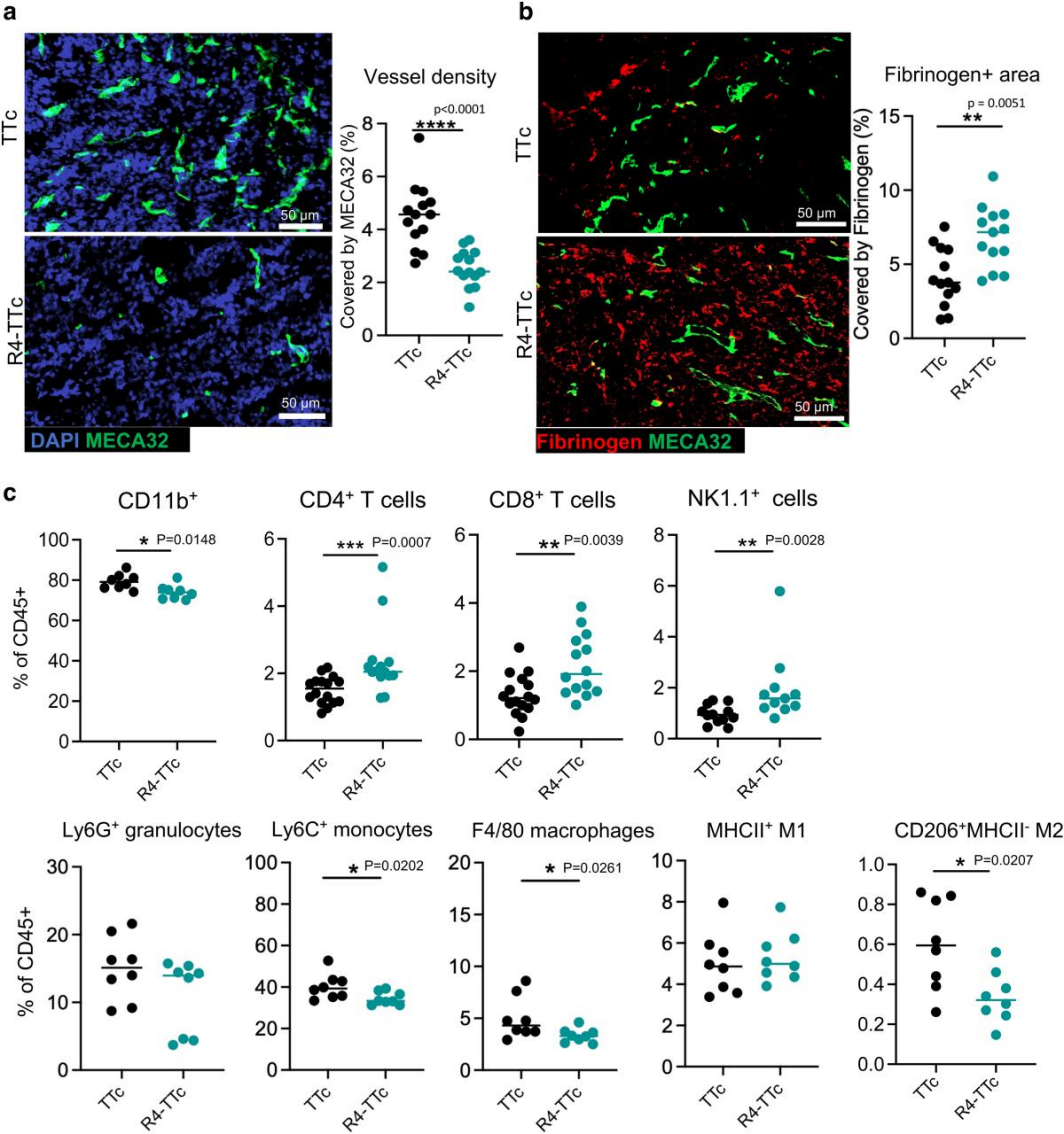
Reduced vascular density and immune cell change in tumours after injection of Robo4-TTc. (a, b) Tissue samples from experiments shown in Figure 4a. Mice were primed with TTc in alum, 3 weeks later were boosted with R4-TTc alum or TTc alum. LLC1 cells were injected s.c. in the right flank of the mouse at d7 after the boost. Tumours were stained with MECA32 endothelial marker and fibrinogen. (a) Immunofluorescent image of tumour stained with MECA32 (green) and DAPI (blue). Scale bar 50 µm. MECA32^+^ vessel density. (b) Immunofluorescent image of tumour stained with fibrinogen (red) and MECA32 (green). Scale bar 50 µm. Percentage of the area covered by Fibrinogen. MECA32^+^ vessels and Fibrinogen area were measured using ImageJ. Each symbol represents one mouse. Two-tailed Mann–Whitney *U* test. (c) Tissue samples from experiments shown in Figure 4E. Mice were primed with TTc alum, 4 weeks later boost with R4-TTc alum or TTc alum. LLC1 cells were injected s.c. at 1 day after the boost. Tumours were harvested for FACS quantification. CD4, CD8, NK1.1^+^ cells, and CD11b^+^ innate cells in tumours were quantified. Data of CD4, CD8, and NK1.1^+^ cells from three independent experiments (similar size of tumours from two groups were selected for FACS staining, *n* = 11 or 14). Data of CD11b^+^ innate cells from two independent experiments (*n* = 8). Two-tailed Mann–Whitney *U* test. Each symbol represents one mouse.

Considering Robo4 is expressed in tumour endothelial cells, we analysed tumours by immunofluorescence histology to understand the effect of Robo4-specific antibody on the tumour vessels. Using tumours from the experiments shown in Fig. 4a, the percentage of MECA32^+^ vessel area on tumour sections was quantified. We found a significant reduction in the vessel density in tumours from the R4-TTc vaccinated group (Fig. 5a). Moreover, tumour sections were stained for fibrinogen, since deposition of this protein is an indicator of increased vascular leakage [32]. This showed significantly increased staining of fibrinogen in R4-TTc vaccinated group (Fig. 5b), indicating increased vessel damage. These results are consistent with changes to tumour vasculature we showed previously [7].

Tumour tissues from experiments shown in Fig. 4e were further analysed by flow cytometry to characterize immune cell populations infiltrating the tumours (Supplementary Fig. S9). Tumours of similar size were selected from the two groups for analysis. As expected, CD11b^+^ myeloid cells were the predominant cell type in LLC1 tumours, with a small population of CD4^+^ T cells and CD8^+^ T cells (around 1% of CD45^+^ cells). The percentage of CD4^+^, CD8^+^ T cells and NK1.1^+^ cells increased in the R4-TTc vaccinated group compared with the control (Fig. 5c top). Vaccination induced a decrease in most CD11b^+^ phagocytic cells. Most notably, the percentage of CD206^+^ MHCII^−^ M2-like tumour-associated macrophages (TAMs) was reduced (Fig. 5c bottom). A recent study suggests M2-like TAMs have immunosuppressive effects, resulting in resistance to chemotherapy and immune checkpoint therapies (ICT). Changes in these immune cell proportions, particularly loss of CD11b^+^ cells, may indicate a transition from a suppressive tumour environment to antitumour immunity after R4-TTc vaccination [33].

We previously showed that the Robo4-specific immune response does not affect wound healing or induce other adverse effects [7]. Vaccinated mice in this study did not display any obvious side effects (e.g. aberrant behaviour, potential weight loss, local side-effects, etc.) to R4-TTc alum immunizations. Further, histochemical microscopy of various organs (heart, kidney, spleen, liver, and pancreas) showed no observable differences regarding morphology or vasculature in R4-TTc and TTc immunized mice (Supplementary Fig. S10). All results suggest that vaccination against Robo4 has no major side effects on organ integrity, making the R4-TTc vaccination strategy as a potential therapeutic intervention for human patients.

### Robo4-TTc vaccination restricts 4T1 tumour growth

**Figure 6:**
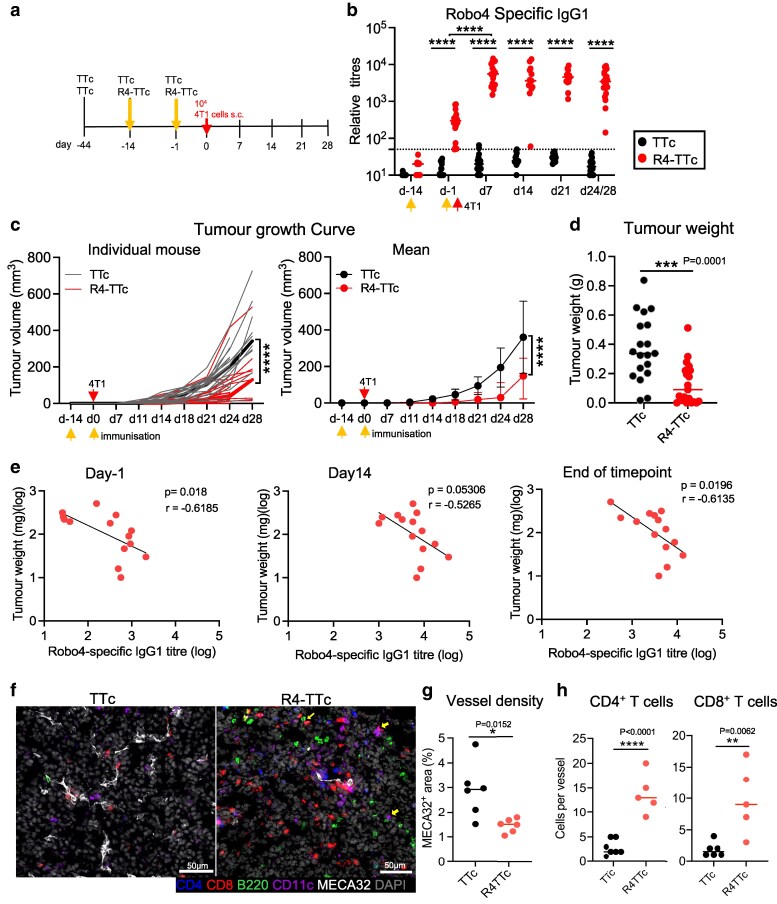
Reduced tumour growth after injection of Robo4-TTc. (a) Experiment protocol. Mice were primed with TTc in alum, 4 weeks later boost with R4-TTc in alum or TTc in alum. 4T1 cells were s.c. injected into the 4th mammary fat pad 1 day after the second boost with R4-TTc in alum or TTc in alum. (b) Robo4-specific IgG1 antibody after the R4-TTc injection. Dash line: detection limit. Two-way ANOVA with mixed-effects analysis (Tukey multiple comparison); ****P < 0.0001. (c) Tumour growth curve. Left: Individual tumour growth curve. Bold line indicates mean. Right: Same data plotted as mean ± SD. Two-way ANOVA with mixed-effects analysis; ****P < 0.0001. (d) Tumour weight at death. Two-tailed Mann–Whitney *U* test. (e) Correlation between the Robo4-specific IgG1 at d-1 (when tumour cells were transplanted), day 14, and the end timepoint with final tumour weight. Two-tailed compute Pearson correlation coefficients. (b–e) Each dot represents one animal, merged two independent experiments. (f) Representative immunofluorescent images of tumours stained with MECA32, CD4, CD8, B220, CD11C, and DAPI. Scale bar: 50 µm. (g) MECA32^+^ vessel density. (h) Immune cell infiltration. Left: CD4^+^ T cells, right: CD8^+^ T cells. Cells quantified within a 100 μm radius surrounding tumour-associated blood vessels (MECA32^+^). (g–h): Each dot represents one animal. Two-tailed Mann–Whitney *U* test.

We wanted to test whether this R4-TTc vaccine strategy has broad applicability across various tumour types. The 4T1 tumour model is a triple negative breast cancer (TNBC), and similar to LLC1 tumours, it is known for responding poorly to immune checkpoint therapies (ICT) [34]. Targeting angiogenesis presents a potential therapy for TNBC [35, 36]. Our data suggest that the amount of Robo4-specific antibody is important for inhibiting tumour growth. Mice were primed with TTc in alum and received 4T1 tumour cells one day after the second R4-TTc in alum boost (Fig. 6a). For further analysing immune cell infiltration in tumour tissue, most tumours from both groups were harvested at the same time at d24 and d28 before reaching the severity limitation. Robo4-specific antibodies were detected 14 days after the first R4-TTc in alum boost (Fig. 6b). By d7 (1-week post-second immunization), Robo4-specific antibody titres increased significantly, reaching their peak. Tumours were detectable approximately 10 days post-transplantation. Tumour growth in the R4-TTc group was significantly delayed compared with controls (Fig. 6c–d). Correlation analyses between the final tumour weights and Robo4-specific IgG1 antibody titres at different time points showed a significant negative correlation for Robo4-specific titres, suggesting Robo4 specific antibody can delay the tumour growth (Fig. 6e, Supplementary Fig. S11). Furthermore, MECA32 immunofluorescence staining showed MECA32^+^ vessel area was significantly reduced in tumours from the R4-TTc group compared with controls (Fig. 6f, g). We further assessed the spatial distribution of immune cells in tumour sections within a 100 μm radius surrounding blood vessels (MECA32^+^) [37]. Interestingly, a major increase in CD4^+^ and CD8^+^ T cells was detected near tumour vasculature in R4-TTc vaccinated group (Fig. 6h). These findings suggest that the R4-TTc vaccination slows down 4T1 tumour growth, is associated with a reduction in tumour vascularization, and promotes CD4 and CD8 T cell infiltration.

## Discussion

Conjugate vaccines were developed decades ago to overcome the poor immunogenicity of capsular polysaccharide antigens in children and aged people. B cells binding purified polysaccharides are not able to recruit T cell help, while conjugate vaccines efficiently activate T and B cell responses inducing affinity maturation and immunological memory [38] [10]. Hapten molecules such as NP mimic polysaccharides, being unable to recruit T cell help and have been used extensively to reveal the mechanism of conjugate responses. Here, the same carrier KLH in alum priming could induce a strong immune response to poorly immunogenic antigen hapten by inducing carrier-specific CD4 T cells, which can provide the necessary ‘help’ for the expansion and differentiation of NP specific B-lymphocytes [26, 27]. Similarly, self-reactive B cells (or anergic B cells) cannot respond to the self-antigen without T cell help, and therefore a carrier will be selected to recruit T cells [18]. Carrier specific CD4 T cells and pre-existing carrier specific antibody could support self-reactive B cells responding to self-antigen. Non-toxic fragment of Tetanus Toxin (TTc) was chosen as a carrier protein, as it is safely used in humans and most patients have specific immunity [12, 39]. Previous research suggests that an ideal carrier should not be a strong antigen, but a molecule that can recruit CD4 T cell help while is unable to induce a significant immune response to itself [40]. We genetically linked the extracellular domain of mouse Robo4 to TTc. This recombinant R4-TTc protein has the advantages of defined components, high safety and easy scalability. Our results show that carrier priming and boost with R4-TTc in alum elicits a strong Robo4-specific IgG1 antibody response. Several studies have shown that this carrier prime-boost strategy induced an enhanced polysaccharide-specific antibody response [41, 42], suggesting the induction of carrier-specific T cell memory is important [26].

Robo4 was identified as a potential vaccine target due to its selective expression in tumour vessels and its presence in a variety of tumours [4, 6, 43]. Robo4-TTc recombinant protein in alum can induce Robo4 specific IgG1 production and delay tumour growth. The tumour size in LLC1 models is negatively correlated with Robo4 specific IgG1 titre, which suggests antibody induced by Robo4-TTc vaccination alone could control tumour growth (Fig. 4g–i), and has therapeutic potential in LLC1 tumours. Our previous study showed that the Robo4-CGG vaccination alone could retard LLC1 tumour growth, and its efficiency is independent of CD8 T cells, but requires B cells and IgG1 [7], supporting that the primary mechanism of tumour suppression in this vaccination strategy is likely antibody-mediated. Furthermore, no side-effect was observed in R4-TTc in alum vaccinated group. Robo4 knock-out mice are viable, fertile, and show normal embryonic development of blood vessels, remain healthy through their lifetime, which indicates that Robo4 is not required for fundamental angiogenesis during early mouse embryogenesis [44]. But further pre-clinic experiments will be designed to assess the long-term effect of Robo4 specific antibody on vascular development, particularly during the pregnancy.

MECA32^+^ vessels significantly decreased in tumours in the Robo4-TTc treated group compared with the control TTc group. Furthermore, our data show an increased percentage of T cells and NK1.1 cells, and a decreased percentage of CD11b^+^Ly6C^−^F4/80^+^ CD206^+^MHCII^−^ M2 like TAMs in tumours after vaccination. These T cells appeared in close proximity other cells and were closely associated with blood vessels in R4-TTc in alum vaccinated tumours (Fig. 6f–h), which may contribute to enhanced antitumour immunity. In contrast, tumours from the control TTc group exhibited higher vessel density and lower immune cell infiltration, with immune cells appearing more dispersed across the section rather than in close contact. This disorganization and poor integrity likely create an inefficient system for immune cell trafficking from the bloodstream into the tumour parenchyma [45]. These results suggest that the vaccination strategy, by disrupting abnormal tumour vessel development, may normalize the tumour microenvironment and lead towards a more immunostimulatory profile with an increased proportion of T cells, NK cells, and loss of CD11b^+^ cells. This could contribute to overall tumour control.

It may be possible to combine this conjugate vaccine approach with immune checkpoint inhibitors (like anti-PD-L1/PD-1) to improve the treatment of immune checkpoint therapy (ICT) resistant tumours. Recent pre-clinical and clinical studies suggest that inhibitors of VEGF (vascular endothelial growth factor)/VEGFR2 combined with checkpoint inhibitors can improve response rates with a potential mechanism of action being enhanced vessel normalization leading to tumour infiltration by lymphocytes and the restoration of an anti-tumour environment [37, 46, 47]. Our data indicate that this conjugated vaccine strategy has the potential to improve the results of cancer therapy for ICT-resistant tumours.

## Materials and methods

### Mice and immunization

All experiments were performed on female and male mice, aged 8–12 weeks. C57BL/6j or Balb/c mice were purchased from Harlan Laboratories. S1pr2ERT2^Cre^ mice were kindly provided by T. Kurosaki, RIKEN Center for Integrative Medicine [24]. S1pr2ERT2^Cre/wt^Ai14 were generated by crossing S1pr2ERT2^Cre^ mice with B6.Cg-*Gt(ROSA)26Sortm14(CAG-tdTomato)Hze*/J (007914, Jackson Lab) [25]. Mice were housed in individually ventilated cages in a University of Birmingham Biological Services Unit. The animal research adheres to the ARRIVE guidelines. All procedures on animals were approved by the University of Birmingham Ethics Committee (AWERB, Animal Welfare Ethical Review Board) and performed in accordance with UK Home Office licenses PP8702596 and PP3338444.

NP (4-hydroxy-3-nitrophenylacetyl) (BioSearch Technologies, USA) was conjugated to KLH (keyhole limpet hemocyanin) (Merck) at a ratio of NP_27_-KLH (in-house made). Mice were primed with 50 µg of 9% alum precipitated KLH or ovalbumin (OVA) (Merck) via intraperitoneal (i.p.) injection, and 4 weeks later were immunized with 50 µg soluble NP-KLH or NP-OVA (BioSearch Technologies, USA) via i.p. injection. Alum-precipitated protocol has been described previously [48]. For tracking the memory B cell response, mice were primed with 50 µg of KLH precipitated in alum, and 4 mg of tamoxifen (20 mg/ml in corn oil) (Merck, T2895) was administered at days 5, 8, and 12 via gavage to label GC-derived memory B cells. Four weeks later mice were immunized with 20 µg soluble NP-KLH on one rear foot. For Robo4 vaccination experiments, mice were i.p. primed with 40–50 μg of TTc precipitated in alum, and then 3–4 weeks later, i.p. immunized with 40–50μg of Robo4-TTc precipitated in alum. For some experiments a second boost was administered 2 weeks later.

### Generation of recombinant Robo4-TTc protein

The sequence of the extracellular region of mouse Robo4 (R4) was obtained from Prof. Roy Bicknell [7]. The sequence of TTc was obtained from Dr Natalia Savelyeva [49]. The extracellular domain of mouse Robo4 (R4) was genetically linked to TTc (Supplementary Fig. S1) and stably transfected into HEK293 cells to produce a recombinant R4-TTc fusion protein. The protein was purified from the supernatant of stably transfected HEK293 cells. The generation and purification of the recombinant proteins TTc and R4-TTc are described in Supplementary Materials and Methods (Supplementary Figs S2 and S3).

### Tumour growth experiments

C57BL/6 mice (mixed sex) were subcutaneously (s.c.) implanted with 2.5 × 10^5^ Lewis Lung Carcinoma (LLC1) cells (Cell Line Service, GmbH; 400263). Female Balb/c mice were s.c. implanted with 2 × 10^4^ 4T1 cells (Cytion, Germany; 300300) on 4th mammary fat pad. Tumour size was measured on the indicated days, and tumour volume was calculated following the formula: length × width^2^ × 0.5.

### ELISA assay for the measurement of antibodies in serum

NP_14_-BSA was used for measuring total NP specific antibody whereas NP_2_-BSA was used to measure the high affinity NP specific antibody. Nunc Maxisorp ELISA plates were coated with 5 µg/ml of either NP-BSA reagent. Recombinant His-tagged mouse Robo4 protein (SinoBiological; 51081-M08H) coated at 1 µg/ml was selected to determine Robo4 specific antibody. Alkaline phosphatase (AP)-conjugated goat anti-mouse IgG1, IgG2a, IgG2b, IgG3 and IgM antibodies (Southern Biotech, Cambridge, UK) were used, and then detected by substrate p-Nitrophenyl phosphate (Sigma; N2770). Absorbance was measured at 405 nm on a SpectraMax ABS Plus plate reader. Absorbance values were plotted on GraphPad Prism version 10.1.0.

### Flow cytometry

Tumour tissues were cut into small pieces and placed in 1 ml of digestion buffer consisting of RPMI1640, 1 mg/ml of Collagenase D (Roche; 11088866001) and 10 μg/ml Deoxyribonuclease I (DNase I) (Sigma; DN25). Mixtures were incubated at 36°C in a shaker for 30 min. After the digestion, the cell suspension was mixed with 10μl of EDTA (500 mM, Sigma-Aldrich; E7889) for 5 min on ice before being filtered. The cell suspensions were treated with CD16/CD32 FcR block (Invitrogen; 14–0161-82) for 15 min in FACS buffer (PBS, 2% v/v FBS, 2 mM EDTA). Next, cells were stained with 50 μl of a cocktail of fluorescence-conjugated antibodies (Supplementary Table S10) diluted in Brilliant Stain Buffer (BD Biosciences; 563794) for 30 min on ice. Then, cells were incubated with Live/Dead Fixable Near-lR Dead Cell Stain (lnvitrogen; L10119). Finally, cells were washed and acquired on Fortessa X20 (BD Biosciences) using FACS Diva software. Data were analysed using FlowJo (BD Biosciences; version 10.8.1).

### Haematoxylin-eosin staining and immunofluorescent staining

Snap-frozen tissues were cut into sections of 6–8 μm, and fixed in ice-cold acetone for 20 min. Haematoxylin-Eosin (H&E) staining was performed as described in Supplementary Table S9. Imaging was performed using the Axio Scan Z1 Slide Scanner (Zeiss) in the bright field setting. Images were processed on ZEN Blue (Zeiss).

For fluorescent staining, the slides were incubated with a blocking buffer containing 10% v/v horse serum for 10 min, and then the slides were loaded with primary antibodies for 1 h, and then washed, and followed with secondary antibodies for 1 h in the dark if it is necessary. Later, the slides were mounted with ProLong Diamond antifade mountant (Life Technologies; P36979). Antibodies can be found in Supplementary Table S10, S11. Images were acquired using the Axio Scan Z1 Slide Scanner (Zeiss), were processed using ZEN Blue (Zeiss) and analysed by ImageJ.

### Gene expression by real-time quantitative PCR (RT-qPCR)

Real-time RT-PCR protocol has been described previously [50]. Briefly, RNA was extracted from frozen tissue sections or frozen tumour cells using the RNeasy Mini Kit (Qiagen, 74104). Real-time PCR was performed on cDNA (RT-PCR) in multiplex with β2-microglobulin as housekeeping gene, and gene expression was normalized to β2-microglobulin gene expression levels. The 2^−ΔΔCT^ method was applied for relative quantification of mRNA levels to tumour cell line. The primers and probe for β2-microglobulin were as follows: 5′-CATACGCCTGCAGAGTTAAGCA-3′, Reverse 5′-ATCACATGTCTCGATCCCAGTAGA-3′, Probe Cy5-CAGTATGGCCGAGCCCAAGACCG-ABHQ2 (SIGMA, UK). IL-4 forward: 5′-GATCATCGGCATTTTGAACGA-3′, Reverse 5′-AGGACGTTTGGCACATCCAT-3′, Probe FAM-TGCATGGCGTCCCTTCTCCTGTG-MethRed, IL-13 Forward 5′-TTGAGGAGCTGAGCAACATCAC-3′, Reserve: 5′-GCGGCCAGGTCCACACT-3′, Probe: FAM-CAAGACCAGACTCCCCTGTGCAACG-BHQ1. IgG1 ST Forward: 5′-CGAGAAGCCTGAGGAATGTGT-3′, Reserve: 5′-GGAGTTAGTTTGGGCAGCAGAT-3′ Probe: FAM-TGGTTCTCTCAACCTGTAGTCCATGCCA-BHQ1. The Robo4 TaqMan gene expression assay (Mm00452963_m1) was purchased from ThermoFisher.

### Statistical analysis

All statistical analysis was performed using GraphPad Prism software (version 10.1.0). An un-paired two-sided Wilcoxon–Mann–Whitney *U* test were selected to compare tumour weights, and immune cells infiltration between two groups. Two-way ANOVA with mixed effect analysis was used to compare tumour growth and antibody response in two groups. A mixed-effects model was used with fixed effects for time, treatment, and their interaction, and a random effect for subject to account for repeated measures. Tukey correction was used for multiple comparisons. All data from independent replicates were included in the statistical analysis; ****P < 0.0001; ***P < 0.001; **P < 0.01; *P < 0.05; ns, not significant. Detailed statistical analysis was described in each figure legend.

## Supplementary Material

ltag005_Supplementary_Data

## Data Availability

Data will be made available on request.

## Permission to reproduce

Graphical abstract Created in BioRender. Escobar-Riquelme F. (2026) https://BioRender.com/na2vhkt

## Abbreviations

TTc: non-toxic fragment C of tetanus toxin
R4: Robo4
R4-TTc: recombinant protein Robo4-TTc
TEM: tumour endothelial marker
LLC1: Lewis Lung Carcinoma
CGG: chicken gamma globulin
KLH: Keyhole limpet haemocyanin
NP: hapten 4-hydroxy-3-nitrophenylacetyl
OVA: chicken ovalbumin
GC: Germinal centre
mBC: memory B cells
Td: tdTomato
MECA32: panendothelial cell antigen
TAM: tumour-associated macrophage
ICT: immune checkpoint therapy
