# Supplementary material for: Vaccination against tumour endothelial marker Robo4 inhibits tumour growth
Source: Immunother Adv. 2026 Jun 25;6(1):ltag005. doi: 10.1093/immadv/ltag005 (PMC13296997; doi:10.1093/immadv/ltag005)
Supplement: ltag005_Supplementary_Data [file ltag005_supplementary_data.zip › Escobar-Riquelme et al Supplementary Tables.pdf]

Supplementary Tables

Suppl Table 1. Commercial and in-house made plasmids

Plasmid name	Description	Source
pUCIDT-R4	Fc-tagged mouse Robo4 sequence in commercial vector pUCIDT, flanked by ApaI and NheI restriction enzymes. Codon optimised for mammalian expression. Bacterial resistance: Ampicillin.	IDT
pUCIDT-TTc	Fc-tagged non-toxic Tetanus toxin fragment C sequence in commercial vector pUCIDT, flanked by ApaI and NheI restriction enzymes. Codon optimised for mammalian expression. Bacterial resistance: Ampicillin.	IDT
pcDNA3.1-mGFP	Vector for mammalian expression of mGFP (membrane GFP) under the CMV promoter. mGFP is flanked by ApaI and NheI restriction enzymes. Bacterial resistance: Ampicillin. Selection marker: Neomycin.	Prepared in-house
pcDNA3.1-R4	In-house construction by restriction enzyme subcloning between ApaI and NheI digested and gel purified pcDNA3.1 mGFP and pUCIDT-R4. Bacterial resistance: Ampicillin. Selection marker: Neomycin.	Prepared in-house
pcDNA3.1-TTc	In-house construction by restriction enzyme subcloning between ApaI and NheI digested and gel purified pcDNA3.1 mGFP and pUCIDT-TTc. Bacterial resistance: Ampicillin. Selection marker: Neomycin	Prepared in-house
pcDNA3.1-R4TTc	In-house construction by PCR cloning and seamless DNA assembly. R4 sequence plus its following linker were PCR amplified from pcDNA3.1-R4 with primers which contained overhangs to match the backbone vector. pcDNA3.1-TTc was PCR linearized with primers which excluded the TTc ATG start codon. Bacterial resistance: Ampicillin. Selection marker: Neomycin.	Prepared in-house

Suppl. Table 2. Fc-tagged mouse Robo4 DNA sequence

GCTAGCGCCACCATGGGCTCAGGGGGACTGGGCTGCTGGGAACCGAATGGCCTCTGCCTCTGCTGCTGCTGTT
TATTATGGGGGAGAAGCTCTGGATTCTCCCCCTCAGATCCTGGTGCACCCACAGGACCAGCTGCTGCAGGGAA
GCGGCCCTGCAAAGATGAGGTGCAGAAGCTCCGGACAGCCACCACCTACCATCAGGTGGCTGCTGAACGGCCAG
CCACTGAGCATGGCCACACCCGACCTGCACTACCTGCTGCCTGATGGCACCCTGCTGCTGCACAGGCCTTCCGT
GCAGGGCCGCCCACAGGACGATCAGAACATCCTGTCCGCCATCCTGGGCGTGTATACATGCGAGGCCTCTAATA
GGCTGGGAACCGCCGTGTCTAGGGGCGCCAGACTGAGCGTGGCCGTGCTGCAGGAGGACTTCCAGATCCAGCCA
AGGGATACAGTGGCAGTGGTGGGAGAGAGCCTGGTGCTGGAGTGTGGCCCACCATGGGGATACCCAAAGCCCTC
TGTGAGCTGGTGGAAGGACGGCAAGCCTCTGGTGCTGCAGCCAGGCCGGAGAACCGTGAGCGGCGACTCCCTGA
TGGTGTCTAGGGCCGAGAAGAATGATAGCGGCACCTATATGTGCATGGCCACAAACAATGCAGGACAGAGGGAG
AGCAGGGCAGCACGGGTGTCCATCCAGGAGTCTCAGGATCACAAGGAGCACCTGGAGCTGCTGGCCGTGCGCAT
CCAGCTGGAGAACGTGACACTGCTGAATCCTGAGCCAGTGAAAGGCCCCAAGCCTGGCCCAAGCGTGTGGCTGT
CCTGGAAGGTGTCTGGCCCTGCAGCACCAGCAGAGAGCTACACCGCCCTGTTCAGAACACAGAGGTCCCCCCGC
GACCAGGGCTCTCCTTGGACCGAGGTGCTGCTGAGGGGCCTGCAGTCCGCCAAGCTGGGAGGCCTGCACTGGGG
ACAGGACTATGAGTTCAAGGTGCGGCCCTCTAGCGGACGGGCCAGAGGCCCCGATAGCAACGTGCTGCTGCTGA
GGCTGCCAGAGCAGGTGCCATCCGCCCCTCCACAGGGAGTGACCCTGCGCAGCGGAAACGGATCCGTGTTCGTG
TCTTGGGCTCCCCCTCCAGCCGAGAGCCACAATGGCGTGATCCGGGGCTACCAAGTGTGGAGCCTGGGAAACGC
CTCCCTGCCAGCAGCCAATTGGACCGTGGTGGGCGAGCAGACACAGCTGGAGATCGCCACCAGACTGCCAGGAT
CCTATTGCGTGCAGGTGGCAGCAGTGACAGGAGCAGGAGCAGGCGAGCTGAGCACCCCCGTGTGCCTGCTGCTG
GAGCAGGCAATGGAGCAGTCCGCCCGGGATCCCAGAAAGCACGTGCCTTGGACACTGGAGCAGCTGAGGGCCAC
CCTGAGGCGCCCAGAGGGAGGACGCGGCTCCTCTAGCTCCGAGCCCAAGTCCTGCGACAAGACCCACACATGCC
CACCTTGTCCAGCACCTGAGCTGCTGGGCGGCCCTAGCGTGTTCCTGTTTCCACCCAAGCCAAAGGATACACTG
ATGATCTCTCGGACCCCCGAGGTGACATGCGTGGTGGTGGACGTGAGCCACGAGGATCCTGAGGTGAAGTTTAA
CTGGTACGTGGACGGCGTGGAGGTGCACAATGCCAAGACCAAGCCCCGGGAGGAGCAGTACAACAGCACATATA
GAGTGGTGTCCGTGCTGACCGTGCTGCACCAGGATTGGCTGAACGGCAAGGAGTACAAGTGCAAGGTGTCCAAT
AAGGCCCTGCCAGCCCCCATCGAGAAGACAATCTCTAAGGCAAAGGGACAGCCAAGGGAGCCTCAGGTGTACAC
CCTGCCTCCATCTAGAGACGAGCTGACAAAGAACCAGGTGAGCCTGACCTGTCTGGTGAAGGGCTTCTATCCTA
GCGATATCGCCGTGGAGTGGGAGTCCAATGGCCAGCCAGAGAACAATTACAAGACCACACCCCCTGTGCTGGAC
TCTGATGGCAGCTTCTTTCTGTATTCCAAGCTGACCGTGGATAAGTCTAGATGGCAGCAGGGCAACGTGTTCAG
CTGCTCTGTGATGCACGAAGCACTGCATAACCACTACACTCAGAAGAGCCTGTCACTGTCACCTGGGAAATAAG
GGCCC

Legend: **NheI** + **Kozac** + **Start codon** + **Robo4** + **Linker1** + **Fc-tag** + **Stop codon** + **ApaI**

Suppl. Table 3. Fc-tagged Fragment C of Tetanus Toxin (TTc) DNA sequence

GCTAGCAAGCTTGCCACCATGGGATGGTCTTGTATTATCTTCTTTCTGGTCGCCACTGCCACTGGGGTCTACTC
AAAAAATCTGGACTGTTGGGTCGATAACGAAGAGGACATCGATGTGATCCTGAAGAAGTCCACAATCCTGAATC
TGGACATCAACAATGATATCATCTCCGACATCTCTGGCTTCAACAGCTCCGTGATCACCTACCCTGACGCCCAG
CTGGTGCCAGGCATCAATGGCAAGGCCATCCACCTGGTGAACAATGAGTCTAGCGAAGTGATCGTGCACAAGGC
CATGGATATCGAGTATAACGACATGTTCAACAACTTCACCGTGAGCTTCTGGCTGCGGGTGCCCAAGGTGTCTG
CCAGCCACCTGGAGCAGTACGGCACCAATGAGTATTCCATCATCTCCTCTATGAAGAAGCACTCCCTGTCTATC
GGCTCTGGCTGGAGCGTGTCCCTGAAGGGCAACAATCTGATCTGGACACTGAAGGATAGCGCCGGAGAGGTGCG
GCAGATCACCTTCCGGGACCTGCCTGATAAGTTTAATGCCTACCTGGCCAACAAGTGGGTGTTCATCACCATCA
CAAACGACCGGCTGAGCTCCGCCAATCTGTATATCAACGGCGTGCTGATGGGCTCTGCCGAGATCACAGGCCTG
GGAGCCATCAGGGAGGATAACAATATCACCCTGAAGCTGGACCGCTGCAACAATAACAATCAGTACGTGAGCAT
CGATAAGTTCAGAATCTTTTGTAAGGCCCTGAATCCTAAGGAGATCGAGAAGCTGTACACCAGCTATCTGTCCA
TCACATTCCTGAGGGATTTTTGGGGCAACCCACTGCGCTACGACACCGAATACTATCTGATCCCCGTGGCCTCT
AGCTCCAAGGATGTGCAGCTGAAGAATATCACCGACTACATGTATCTGACAAACGCCCCTTCCTATACCAATGG
CAAGCTGAACATCTACTATCGGAGACTGTACAACGGCCTGAAGTTCATCATCAAGCGGTATACCCCAAACAATG
AGATCGATTCCTTCGTGAAGTCTGGCGACTTTATCAAGCTGTACGTGAGCTATAACAATAACGAGCACATCGTG
GGCTACCCAAAGGATGGCAATGCCTTTAATAACCTGGACAGAATCCTGAGGGTGGGATACAACGCACCAGGCAT
CCCTCTGTATAAGAAGATGGAGGCCGTGAAGCTGAGGGACCTGAAGACATACTCCGTGCAGCTGAAGCTGTATG
ACGATAAGAATGCCTCTCTGGGCCTGGTGGGCACCCACAACGGACAGATCGGCAATGATCCCAACCGCGACATC
CTGATCGCCAGCAATTGGTACTTCAACCACCTGAAGGATAAGATCCTGGGCTGCGACTGGTATTTTGTGCCAAC
CGATGAGGGCTGGACAAATGACGGAGGCAGCGGAGGAGGCTCCGGAGGAGAGCCCAAGAGCTGTGATAAGACCC
ACACATGCCCACCTTGTCCAGCACCAGAGCTGCTGGGCGGCCCTTCCGTGTTCCTGTTTCCACCCAAGCCTAAG
GACACACTGATGATCAGCAGGACCCCCGAGGTGACATGCGTGGTGGTGGACGTGAGCCACGAGGACCCTGAGGT
GAAGTTCAATTGGTACGTGGATGGCGTGGAGGTGCACAACGCCAAGACCAAGCCCCGGGAGGAGCAGTACAACT
CTACATATAGAGTGGTGAGCGTGCTGACCGTGCTGCACCAGGACTGGCTGAATGGCAAGGAGTACAAGTGCAAG
GTGTCTAACAAGGCCCTGCCTGCCCCAATCGAGAAGACAATCAGCAAGGCAAAGGGACAGCCACGGGAGCCACA
GGTGTACACCCTGCCTCCCAGCCGGGACGAGCTGACAAAGAATCAGGTGAGCCTGACCTGTCTGGTGAAGGGCT
TCTATCCTAGCGACATCGCAGTGGAGTGGGAGTCCAACGGACAGCCAGAGAATAACTACAAGACCACACCCCCT
GTGCTGGACTCTGATGGCAGCTTCTTTCTGTATTCCAAGCTGACCGTGGACAAGTCTCGCTGGCAGCAGGGCAA
CGTGTTTTCTTGTAGCGTGATGCATGAAGCACTGCACAACCATTACACCCAGAAAAGCCTGTCACTGTCCCCCG
GAAAGTAATAGCTCGAGTCTAGAGGGCCC

Legend: **Nhel+ Hindll** + **Kozac** + **Start codon** + **TTc** + **Linker2** + **Fc-tag** + **Stop codons** + **XbaI** + **ApaI** +**XhoI**

Suppl. Table 4. Genetically linked and Fc-tagged R4-TTc DNA sequence

GCTAGCGCCACCATGGGCTCAGGGGGACTGGGCTGCTGGGAACCGAATGGCCTCTGCCTCTGCTGCTGCTGTT
TATTATGGGGGAGAAGCTCTGGATTCTCCCCCTCAGATCCTGGTGCACCCACAGGACCAGCTGCTGCAGGGAA
GCGGCCCTGCAAAGATGAGGTGCAGAAGCTCCGGACAGCCACCACCTACCATCAGGTGGCTGCTGAACGGCCAG
CCACTGAGCATGGCCACACCCGACCTGCACTACCTGCTGCCTGATGGCACCCTGCTGCTGCACAGGCCTTCCGT
GCAGGGCCGCCCACAGGACGATCAGAACATCCTGTCCGCCATCCTGGGCGTGTATACATGCGAGGCCTCTAATA
GGCTGGGAACCGCCGTGTCTAGGGGCGCCAGACTGAGCGTGGCCGTGCTGCAGGAGGACTTCCAGATCCAGCCA
AGGGATACAGTGGCAGTGGTGGGAGAGAGCCTGGTGCTGGAGTGTGGCCCACCATGGGGATACCCAAAGCCCTC
TGTGAGCTGGTGGAAGGACGGCAAGCCTCTGGTGCTGCAGCCAGGCCGGAGAACCGTGAGCGGCGACTCCCTGA
TGGTGTCTAGGGCCGAGAAGAATGATAGCGGCACCTATATGTGCATGGCCACAAACAATGCAGGACAGAGGGAG
AGCAGGGCAGCACGGGTGTCCATCCAGGAGTCTCAGGATCACAAGGAGCACCTGGAGCTGCTGGCCGTGCGCAT
CCAGCTGGAGAACGTGACACTGCTGAATCCTGAGCCAGTGAAAGGCCCCAAGCCTGGCCCAAGCGTGTGGCTGT
CCTGGAAGGTGTCTGGCCCTGCAGCACCAGCAGAGAGCTACACCGCCCTGTTCAGAACACAGAGGTCCCCCCGC
GACCAGGGCTCTCCTTGGACCGAGGTGCTGCTGAGGGGCCTGCAGTCCGCCAAGCTGGGAGGCCTGCACTGGGG
ACAGGACTATGAGTTCAAGGTGCGGCCCTCTAGCGGACGGGCCAGAGGCCCCGATAGCAACGTGCTGCTGCTGA
GGCTGCCAGAGCAGGTGCCATCCGCCCCTCCACAGGGAGTGACCCTGCGCAGCGGAAACGGATCCGTGTTCGTG
TCTTGGGCTCCCCCTCCAGCCGAGAGCCACAATGGCGTGATCCGGGGCTACCAAGTGTGGAGCCTGGGAAACGC
CTCCCTGCCAGCAGCCAATTGGACCGTGGTGGGCGAGCAGACACAGCTGGAGATCGCCACCAGACTGCCAGGAT
CCTATTGCGTGCAGGTGGCAGCAGTGACAGGAGCAGGAGCAGGCGAGCTGAGCACCCCCGTGTGCCTGCTGCTG
GAGCAGGCAATGGAGCAGTCCGCCCGGGATCCCAGAAAGCACGTGCCTTGGACACTGGAGCAGCTGAGGGCCAC
CCTGAGGCGCCCAGAGGGAGGACGCGGCTCCTCTAGCTCCGGATGGTCTTGTATTATCTTCTTTCTGGTCGCCA
CTGCCACTGGGGTCTACTCAAAAAATCTGGACTGTTGGGTCGATAACGAAGAGGACATCGATGTGATCCTGAAG
AAGTCCACAATCCTGAATCTGGACATCAACAATGATATCATCTCCGACATCTCTGGCTTCAACAGCTCCGTGAT
CACCTACCCTGACGCCCAGCTGGTGCCAGGCATCAATGGCAAGGCCATCCACCTGGTGAACAATGAGTCTAGCG
AAGTGATCGTGCACAAGGCCATGGATATCGAGTATAACGACATGTTCAACAACTTCACCGTGAGCTTCTGGCTG
CGGGTGCCCAAGGTGTCTGCCAGCCACCTGGAGCAGTACGGCACCAATGAGTATTCCATCATCTCCTCTATGAA
GAAGCACTCCCTGTCTATCGGCTCTGGCTGGAGCGTGTCCCTGAAGGGCAACAATCTGATCTGGACACTGAAGG
ATAGCGCCGGAGAGGTGCGGCAGATCACCTTCCGGGACCTGCCTGATAAGTTTAATGCCTACCTGGCCAACAAG
TGGGTGTTCATCACCATCACAAACGACCGGCTGAGCTCCGCCAATCTGTATATCAACGGCGTGCTGATGGGCTC
TGCCGAGATCACAGGCCTGGGAGCCATCAGGGAGGATAACAATATCACCCTGAAGCTGGACCGCTGCAACAATA
ACAATCAGTACGTGAGCATCGATAAGTTCAGAATCTTTTGTAAGGCCCTGAATCCTAAGGAGATCGAGAAGCTG
TACACCAGCTATCTGTCCATCACATTCCTGAGGGATTTTTGGGGCAACCCACTGCGCTACGACACCGAATACTA
TCTGATCCCCGTGGCCTCTAGCTCCAAGGATGTGCAGCTGAAGAATATCACCGACTACATGTATCTGACAAACG
CCCCTTCCTATACCAATGGCAAGCTGAACATCTACTATCGGAGACTGTACAACGGCCTGAAGTTCATCATCAAG
CGGTATACCCCAAACAATGAGATCGATTCCTTCGTGAAGTCTGGCGACTTTATCAAGCTGTACGTGAGCTATAA
CAATAACGAGCACATCGTGGGCTACCCAAAGGATGGCAATGCCTTTAATAACCTGGACAGAATCCTGAGGGTGG
GATACAACGCACCAGGCATCCCTCTGTATAAGAAGATGGAGGCCGTGAAGCTGAGGGACCTGAAGACATACTCC
GTGCAGCTGAAGCTGTATGACGATAAGAATGCCTCTCTGGGCCTGGTGGGCACCCACAACGGACAGATCGGCAA
TGATCCCAACCGCGACATCCTGATCGCCAGCAATTGGTACTTCAACCACCTGAAGGATAAGATCCTGGGCTGCG
ACTGGTATTTTGTGCCAACCGATGAGGGCTGGACAAATGACGGAGGCAGCGGAGGAGGCTCCGGAGGAGAGCCC
AAGAGCTGTGATAAGACCCACACATGCCCACCTTGTCCAGCACCAGAGCTGCTGGGCGGCCCTTCCGTGTTCCT
GTTTCCACCCAAGCCTAAGGACACACTGATGATCAGCAGGACCCCCGAGGTGACATGCGTGGTGGTGGACGTGA

GCCACGAGGACCCTGAGGTGAAGTTCAATTGGTACGTGGATGGCGTGGAGGTGCACAACGCCAAGACCAAGCCC
CGGGAGGAGCAGTACAACTCTACATATAGAGTGGTGAGCGTGCTGACCGTGCTGCACCAGGACTGGCTGAATGG
CAAGGAGTACAAGTGCAAGGTGTCTAACAAGGCCCTGCCTGCCCCAATCGAGAAGACAATCAGCAAGGCAAAGG
GACAGCCACGGGAGCCACAGGTGTACACCCTGCCTCCCAGCCGGGACGAGCTGACAAAGAATCAGGTGAGCCTG
ACCTGTCTGGTGAAGGGCTTCTATCCTAGCGACATCGCAGTGGAGTGGGAGTCCAACGGACAGCCAGAGAATAA
CTACAAGACCACACCCCCTGTGCTGGACTCTGATGGCAGCTTCTTTCTGTATTCCAAGCTGACCGTGGACAAGT
CTCGCTGGCAGCAGGGCAACGTGTTTTCTTGTAGCGTGATGCATGAAGCACTGCACAACCATTACACCCAGAAA
AGCCTGTCACTGTCCCCCGGAAAGTAATAGCTCGAGTCTAGAGGGCCC

Legend: **NheI** + **Kozac** + **Start codon** + **Robo4** + **Linker** + **TTc** + **Linker** + **Fc-tag** + **Stop codon** + **XbaI** + **ApaI** + **XhoI**

Suppl.Table 5.Fc-tagged Fragment C of Tetanus Toxin (TTc) protein sequence

MGWSCIIFFLVATATGVYSKNLDCWVDNEEDIDVILKKSTILNLDINNDIISDISGFNSSVITYPDAQLVPGIN
GKAIHLVNNESSEVIVHKAMDIEYNDMFNNFTVSFWLRVPKVSASHLEQYGTNEYSIISSMKKHSLSIGSGWSV
SLKGNNLIWTLKDSAGEVRQITFRDLPDKFNAYLANKWVFITITNDRLSSANLYINGVLMGSAEITGLGAIRED
NNITLKLDRCNNNNQYVSIDKFRIFCKALNPKEIEKLYTSYLSITFLRDFWGNPLRYDTEYYLIPVASSSKDVQ
LKNITDYMYLTNAPSYTNGKLNIYYRRLYNGLKFIIKRYTPNNEIDSFVKSGDFIKLYVSYNNNEHIVGYPKDG
NAFNNLDRILRVGYNAPGIPLYKKMEAVKLRDLKTYSVQLKLYDDKNASLGLVGTHNGQIGNDPNRDILIASNW
YFNHLKDKILGCDWYFVPTDEGWTNDGGSGGGSGGEPKSCDKTHTCPPCPAPELLGGPSVFLFPPKPKDTLMIS
RTPEVTCVVVDVSHEDPEVKFNWYVDGVEVHNAKTKPREEQYNSTYRVVSVLTVLHQDWLNGKEYKCKVSNKAL
PAPIEKTISKAKGQPREPQVYTLPPSRDELTKNQVSLTCLVKGFYPSDIAVEWESNGQPENNYKTTPPVLDSDG
SFFLYSKLTVDKSRWQQGNVFSCSVMHEALHNHYTQKSLSLSPGK*

Legend: **TTc** + **Linker** + **Fc-tag**

Suppl. Table 6.Fc-tagged mouse Robo4 protein sequence

MGSGGTGLLGTEWPLPLLLLFIMGGEALDSPPQILVHPQDQLLQGSGPAKMRCRSSGQPPPTIRWLLNGQPLSM
ATPDLHYLLPDGTLLLHRPSVQGRPQDDQNILSAILGVYTCEASNRLGTAVSRGARLSVAVLQEDFQIQPRDTV
AVVGESLVLECGPPWGYPKPSVSWWKDGKPLVLQPGRRTVSGDSLMVSRAEKNDSGTYMCMATNNAGQRESRAA
RVSIQESQDHKEHLELLAVRIQLENVTLLNPEPVKGPKPGPSVWLSWKVSGPAAPAESYTALFRTQRSPRDQGS
PWTEVLLRGLQSAKLGGLHWGQDYEFKVRPSSGRARGPDSNVLLLRLPEQVPSAPPQGVTLRSGNGSVFVSWAP
PPAESHNGVIRGYQVWSLGNASLPAANWTVVGEQTQLEIATRLPGSYCVQVAAVTGAGAGELSTPVCLLLEQAM
EQSARDPRKHVPWTLEQLRATLRRPEGGRGSSSSEPKSCDKTHTCPPCPAPELLGGPSVFLFPPKPKDTLMISR
TPEVTCVVVDVSHEDPEVKFNWYVDGVEVHNAKTKPREEQYNSTYRVVSVLTVLHQDWLNGKEYKCKVSNKALP
APIEKTISKAKGQPREPQVYTLPPSRDELTKNQVSLTCLVKGFYPSDIAVEWESNGQPENNYKTTPPVLDSDGS
FFLYSKLTVDKSRWQQGNVFSCSVMHEALHNHYTQKSLSLSPGK*

Legend: Robo4 + Linker + Fc-tag

Suppl. Table 7.Genetically linked and Fc-tagged R4-TTc protein sequence

MGSGGTGLLGTEWPLPLLLLFIMGGEALDSPPQILVHPQDQLLQGSGPAKMRCRSSGQPPPTIRWLLNGQPLSM
ATPDLHYLLPDGTLLLHRPSVQGRPQDDQNILSAILGVYTCEASNRLGTAVSRGARLSVAVLQEDFQIQPRDTV
AVVGESLVLECGPPWGYPKPSVSWWKDGKPLVLQPGRRTVSGDSLMVSRAEKNDSGTYMCMATNNAGQRESRAA
RVSIQESQDHKEHLELLAVRIQLENVTLLNPEPVKGPKPGPSVWLSWKVSGPAAPAESYTALFRTQRSPRDQGS
PWTEVLLRGLQSAKLGGLHWGQDYEFKVRPSSGRARGPDSNVLLLRLPEQVPSAPPQGVTLRSGNGSVFVSWAP
PPAESHNGVIRGYQVWSLGNASLPAANWTVVGEQTQLEIATRLPGSYCVQVAAVTGAGAGELSTPVCLLLEQAM
EQSARDPRKHVPWTLEQLRATLRRPEGGRGSSSSGWSCIIFFLVATATGVYSKNLDCWVDNEEDIDVILKKSTI
LNLDINNDIISDISGFNSSVITYPDAQLVPGINGKAIHLVNNESSEVIVHKAMDIEYNDMFNNFTVSFWLRVPK
VSASHLEQYGTNEYSIISSMKKHSLSIGSGWSVSLKGNNLIWTLKDSAGEVRQITFRDLPDKFNAYLANKWVFI
TITNDRLSSANLYINGVLMGSAEITGLGAIREDNNITLKLDRCNNNNQYVSIDKFRIFCKALNPKEIEKLYTSY
LSITFLRDFWGNPLRYDTEYYLIPVASSSKDVQLKNITDYMYLTNAPSYTNGKLNIYYRRLYNGLKFIIKRYTP
NNEIDSFVKSGDFIKLYVSYNNNEHIVGYPKDGNAFNNLDRILRVGYNAPGIPLYKKMEAVKLRDLKTYSVQLK
LYDDKNASLGLVGTHNGQIGNDPNRDILIASNWYFNHLKDKILGCDWYFVPTDEGWTNDGGSGGGSGGEPKSCD
KTHTCPPCPAPELLGGPSVFLFPPKPKDTLMISRTPEVTCVVVDVSHEDPEVKFNWYVDGVEVHNAKTKPREEQ
YNSTYRVVSVLTVLHQDWLNGKEYKCKVSNKALPAPIEKTISKAKGQPREPQVYTLPPSRDELTKNQVSLTCLV
KGFYPSDIAVEWESNGQPENNYKTTPPVLDSDGSFFLYSKLTVDKSRWQQGNVFSCSVMHEALHNHYTQKSLSL
SPGK*

Legend: Robo4 + Linker + TTc + Linker + Fc-tag

Suppl. Table 8. Theoretical molecular weight

Molecule	Molecular weight (kDa)
Fc-tagged R4	77.8
R4	50.8
Fc-tagged TTc	80.3
TTc	53.6
Fc-tagged R4TTc	132
Fc-tag	26

Suppl Table 9. Steps for Haematoxylin and Eosin staining

Step	Solution	Time
1	Water	2 min
2	Water	2 min
3	Haematoxylin (HARRIS)	10 min
4	Water	2 min
5	Acid Alcohol	15 s
6	Water	2 min
7	Scott's Tap Water	30 s
8	Water	2 min
9	Eosin	4 min
10	Water	2 min
11	Water	2 min
12	Alcohol	2 min
13	A3 - Alcohol	2 min
14	A2 - Alcohol	2 min
15	A1 - Alcohol	2 min
16	C3 - Xylene	2 min
17	C2 - Xylene	2 min
18	C1 - Xylene	2 min

Haematoxylin (HARRIS): Merck, Cat: HHS32-1L
Eosin: Merck, Cat 318906-500ml
Alcohol: Industrial denatured alcohol 99% (IDA99), UN1170, pfmmedical, lot: 24990, ref:PRC/R/101.
Xylene: UN1370, pfmmedical Lot: 24633, ref:PRC/R/201

Suppl Table10 Primary Antibody list

Specificty	Clone	Source	Reference	RRID	Conjugation	Dilution
Panendothelia cell antigen	MECA32	BD Biosiences	550563		Purified	1/400 IHC
Robo4	Polyclonal	Abcam	ab103674		Purified	1/200 IHC
Fibrinogen	Polyclonal	Abcam	ab36249		Purified	1/500 IHC
Rabbit Polyclonal IgG	Polyclonal	BioLegend	910801		Purified	1/1500 IHC
Robo4	Polyclonal	SinoBiological	51081-T16		Purified	1/5000 blot
human IgG (Fc specific)	GG-7	SIGMA	I6260-2ml		Purified	1/10000 blot
Tetanus Toxoid	TH-11	abcam	ab278064		Purified	1/500 blot
CD45 AF700	30-F11	BioLegend	103128	AB_493714	Purified	1/500 FACS
CD11b	M1/70	BioLegend	101245	AB_2561390	BV510	1/300 FACS
CD11c	HL3	BD Biosiences	558079	AB_10611859	PE-Cy7	1/500 FACS
CD3	17A2	BD Biosiences	740268	AB_2744387	BUV395	1/300 FACS
NK1.1	PK136	eBioscience	12-5941-82	AB_466050	PE	1/500 FACS
CD4	RM4-5	BioLegend	100550	AB_11218995	BV5605	1/500 FACS
CD8a	53-6.5	eBioscience	17-0081-82	AB_469335	APC	1/400 FACS
CD44	IM7	eBioscience	45-0041-82	AB_925746	PerCP	1/300 FACS
CD69	H1.2F3	BioLegend	104530	AB_2562306	BV605	1/300 FACS
CD127	A7R34	BioLegend	135035	AB_11219191	BV711	1/300 FACS
PD1	EH12.2H7	BioLegend	329920	AB_10900818	BV421	1/200 FACS
PDL1	MIH3	BioLegend	374508	AB_2734435	BV421	1/200 FACS
Ly6G	1A8	BioLegend	127605	AB_1236488	FITC	1/500 FACS
Ly6C	HK1.4	BioLegend	128028	AB_10900235	PerCP	1/300 FACS
F4/80	BM8	BioLegend	123133	AB_2562305	BV605	1/400 FACS
CD103	2.00E+07	BioLegend	121406	AB_535948	PE	1/400 FACS
MHCII (1-Ab)	AF6-120.1	eBiosiences	12-5320-82	AB_2572619	APC	1/2000 FACS
CD206	C068C2	BioLegend	141717	AB_2562232	BV421	1/200 FACS
B220	RA3-6B2	BD Biosiences	563793	AB_2738427	BUV395	1/1000 FACS
CD19	1D3	BD Biosiences	612781	AB_2870111	BUV737	1/1000 FACS
CD86	GL1	eBioscience	25-0862-82	AB_2573372	PE-Cy7	1/500 FACS
B220	RA3-6B2	BioLegend	103206	AB_312991	FITC	1/100 IHC
CD4	RM4-5	BioLegend	100516	AB_312719	APC	1/100 IHC
CD8	53-6.5	ebioscience	12-0081-82	AB_465530	PE	1/100 IHC
CD11c	N418	BioLegend	117310	AB_313779	APC	1/100 IHC
CD138	281-2	BioLegend	142519	AB_2562571	BV711	1/500 FACS
CD38	90	BD Biosiences	741748	AB_2871114	BUV737	1/500 FACS
CD95 (Fas)	Jo2	BD Biosiences	740367	AB_2740099	BV605	1/500 FACS
NP		Biosearch Technologies	N-1021-100		APC conjugated in house	1/500 FACS

Suppl Table11 Secondary reagents

Target	Host		Source	Cat	Conjugated	Dilution
Rabbit IgG	Donkey	polyclonal	Jackson Immunoresearch	711-165-152	Cy3	1/500 IHC
Rat IgG	Donkey	polyclonal	Jackson Immunoresearch	712-545-153	Alexa Fluor 488	1/500 IHC
Rabbit IgG	Donkey	polyclonal	Abcam	ab216779	IRDye680RD	1/15000 blot
Mouse IgG	Goat	polyclonal	Abcam	ab216772	IRDye800CW	1/15000 blot
