# Supplementary material for: Vaccination against tumour endothelial marker Robo4 inhibits tumour growth
Source: Immunother Adv. 2026 Jun 25;6(1):ltag005. doi: 10.1093/immadv/ltag005 (PMC13296997; doi:10.1093/immadv/ltag005)
Supplement: ltag005_Supplementary_Data [file ltag005_supplementary_data.zip › Escobar-Riquelme Suppl Figures.pdf]

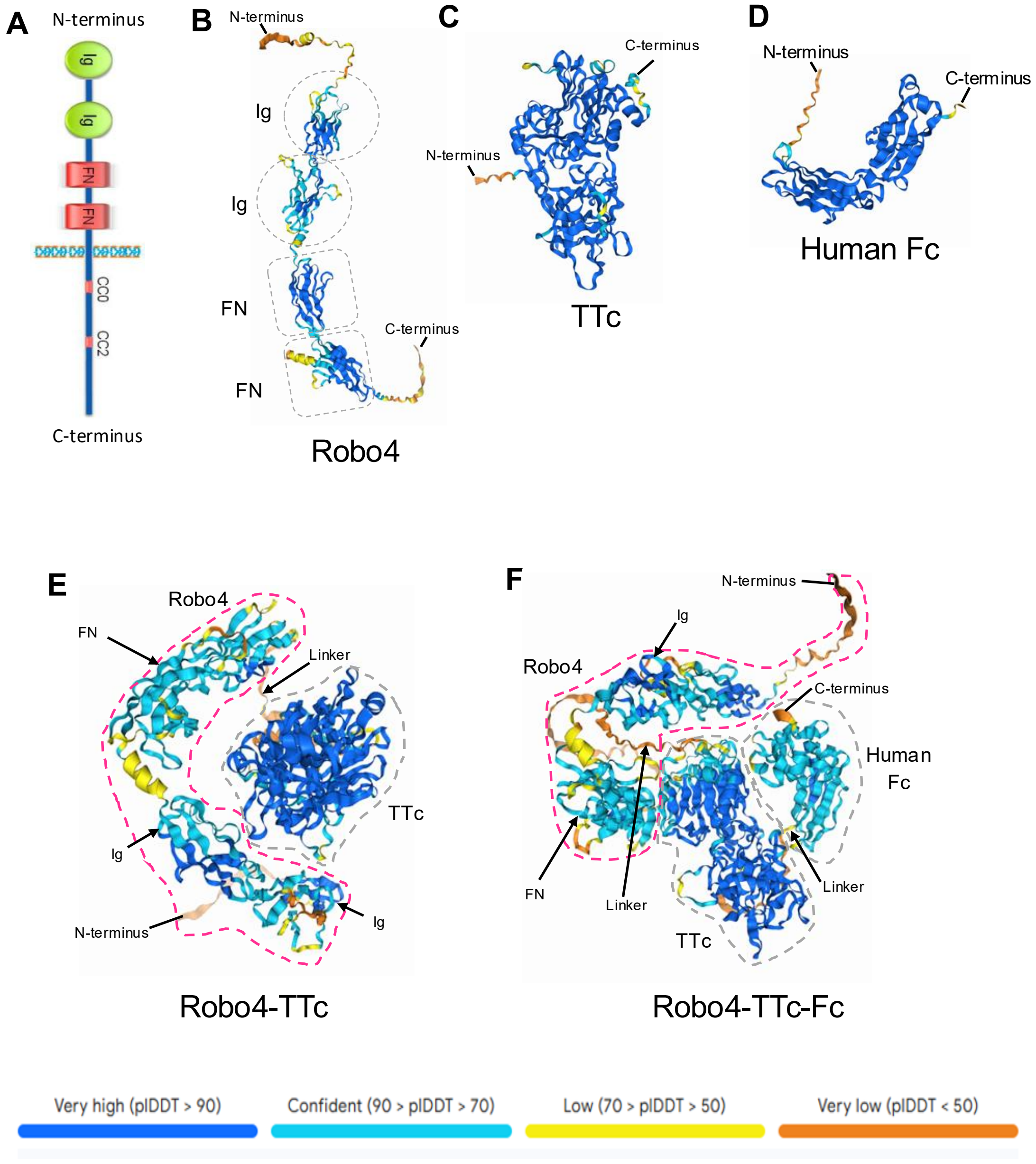

Suppl Figure S1. Structure of the Robo4 proteins.

A) Robo4 receptor structure, Robo4 comprises two immunoglobulin (Ig) and two fibronectin (FN) domains in the extracellular region.

Adapted from Zhuang's thesis [Zhuang, Xiaodong (2013)]. *Validation and identification of tumour endothelial markers and their uses in cancer vaccine.* University of Birmingham. https://etheses.bham.ac.uk/id/eprint/4245/).

B, C, D, E, and F) Predicts 3D structures of Robo4, TTc, Human Fc, Robo4-TTc, and Robo4-TTc-Fc generated using AlphaFold. The images illustrate the predicted folding and linker positioning by displaying the detailed spatial arrangements of each domain and the fusion constructs.

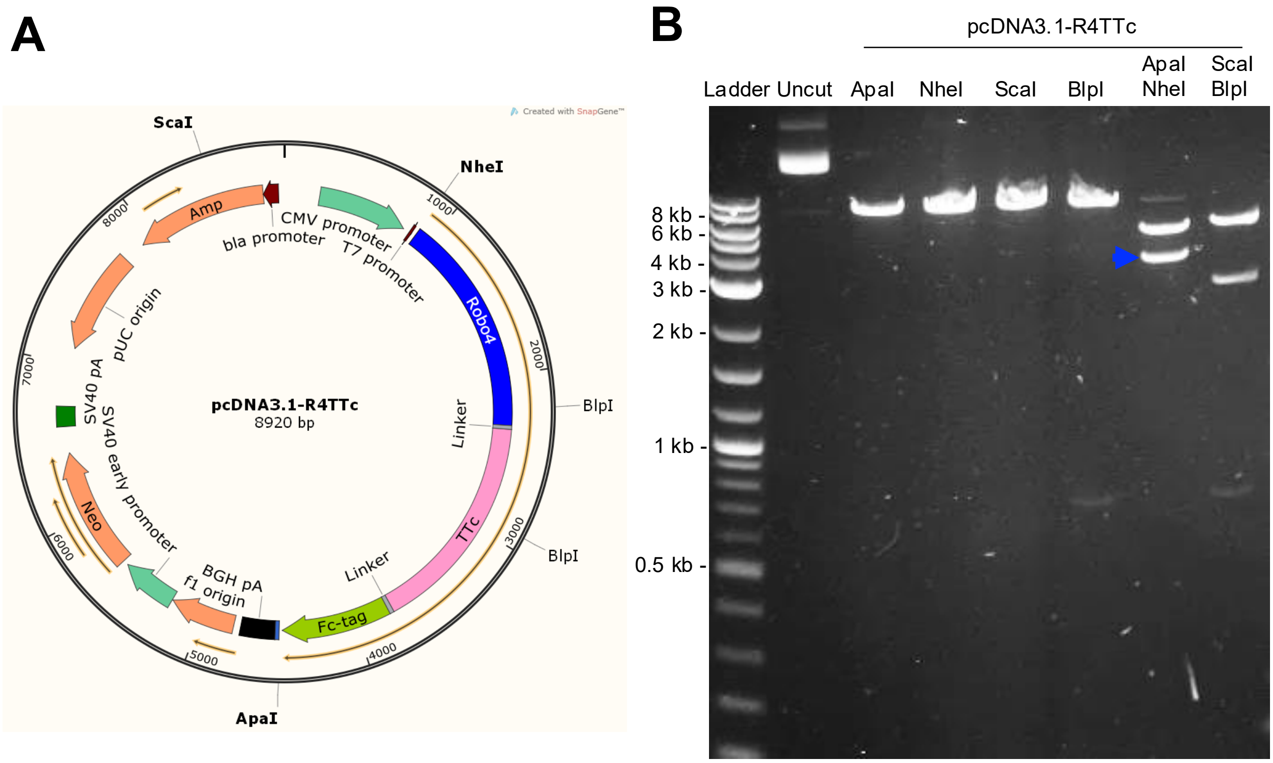

C pcDNA3.1-R4TTc

1. Upstream region

Expected

5' AAGCTGGCTAGCGCCACCATGGGCTCAGGGGGGACTGGG 3'

Sequenced

5' AAGCTGGCTAGCGCCACCATGGGCTCAGGGGGGACTGGG 3'

2. Linker 1

Expected

5' CGCCCAGAGGGAGGACGCGGCTCCTCTAGCTCCGGATGG 3'

Sequenced

5' CGCCCAGAGGGAGGACGCGGCTCCTCTAGCTCCGGATGG 3'

Legend:

1. pcDNA3.1 + NheI + Kozac + Start codon + Robo4
2. Robo4 + Linker 1 + TTc

3. Linker 2

Expected

5' ACAAATGACGGAGGCAGCGGAGGAGGCTCCGGAGGAGAGCCC 3'

Sequenced:

5' ACAAATGACGGAGGCAGCGGAGGAGGCTCCGGAGGAGAGCCC 3'

4. Downstream region

Expected

5' ACTGTCCCCCGGAAAGTAATAGCTCGAGTCTAGAGGGCCC 3'

Sequenced

5' ACTGTCCCCCGGAAAGTAATAGCTCGAGTCTAGAGGGCCC 3'

Legend:

3. TTc + Linker 2 + Fc-tag
4. Fc-tag + Stop codons + XbaI + ApaI + XhoI

Suppl Figure S2. pcDNA3.1-R4TTc assembly analysed by restriction enzyme digestion and sequencing.

A) pcDNA3.1-R4TTc vector map designed on SnapGene. **B**) Visualization of pcDNA3.1-R4TTc fingerprint digestion with different enzymes. Blue arrow shows R4-TTc-Fc fragment. **C**) pcDNA3.1-R4TTc representative results of sanger sequencing of the upstream, downstream and linker regions compared to their expected sequences.

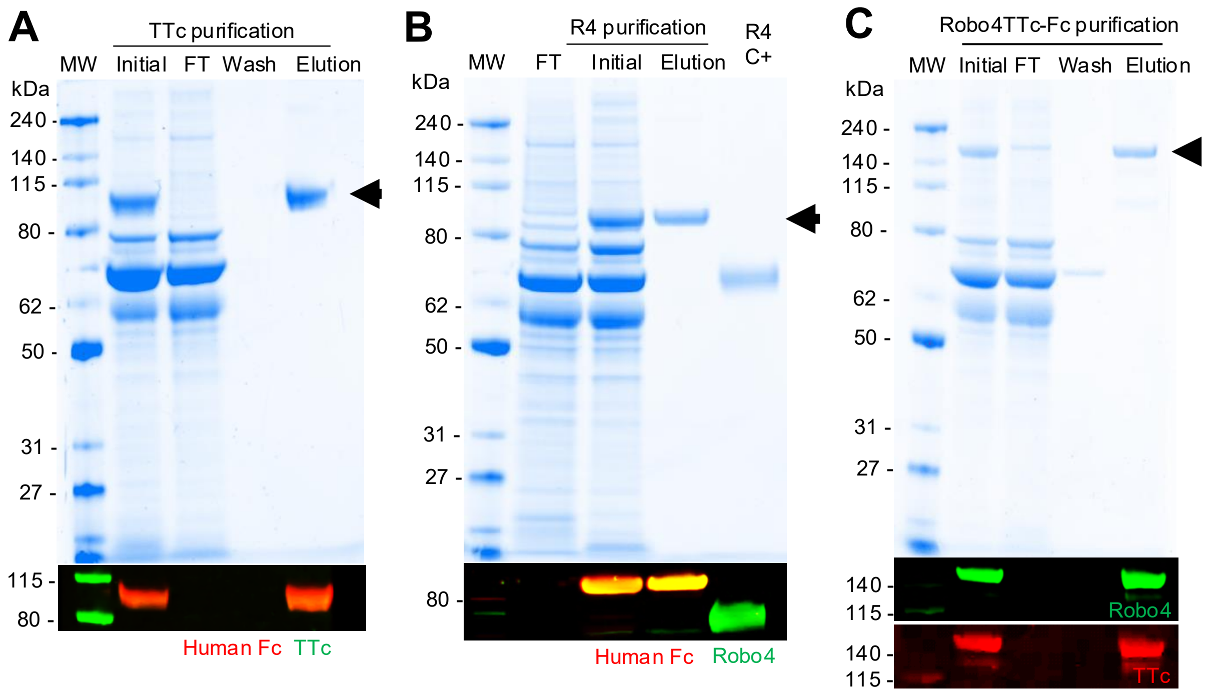

Suppl Figure S3. Genetically engineered protein Robo4 with TTc

Supernatant of stably transfected cells expressing TTc protein, Robo4 protein, and R4-TTc protein were purified, and analysed by SDS-PAGE and western blot. “Initial”: Supernatant before purification. “FT”: Flow-through (non-bound). “Wash”: PBS for washing column (Protein A HP). “Elusion”: collection of elution by adding acid medium into the column. SDS-PAGE and western blot of the purification of **A**) TTc-Fc, **B**) Robo4-Fc, **C**) R4TTc-Fc protein. TTc protein was detected with an anti-TTc antibody (green) and an anti-human Fc (red). The protein has a MW of 80.3 kDa. The Robo4 protein and Fc-tag were detected using specific antibodies against Robo4 and the human-Fc, “R4 C+” is a commercial Robo4 His tagged protein used as a positive control. The Robo4-Fc protein is 77.8kDa. The R4-TTc protein was detected with the anti-Robo4 and anti-TTc antibodies. The expected MW is 132 kDa.

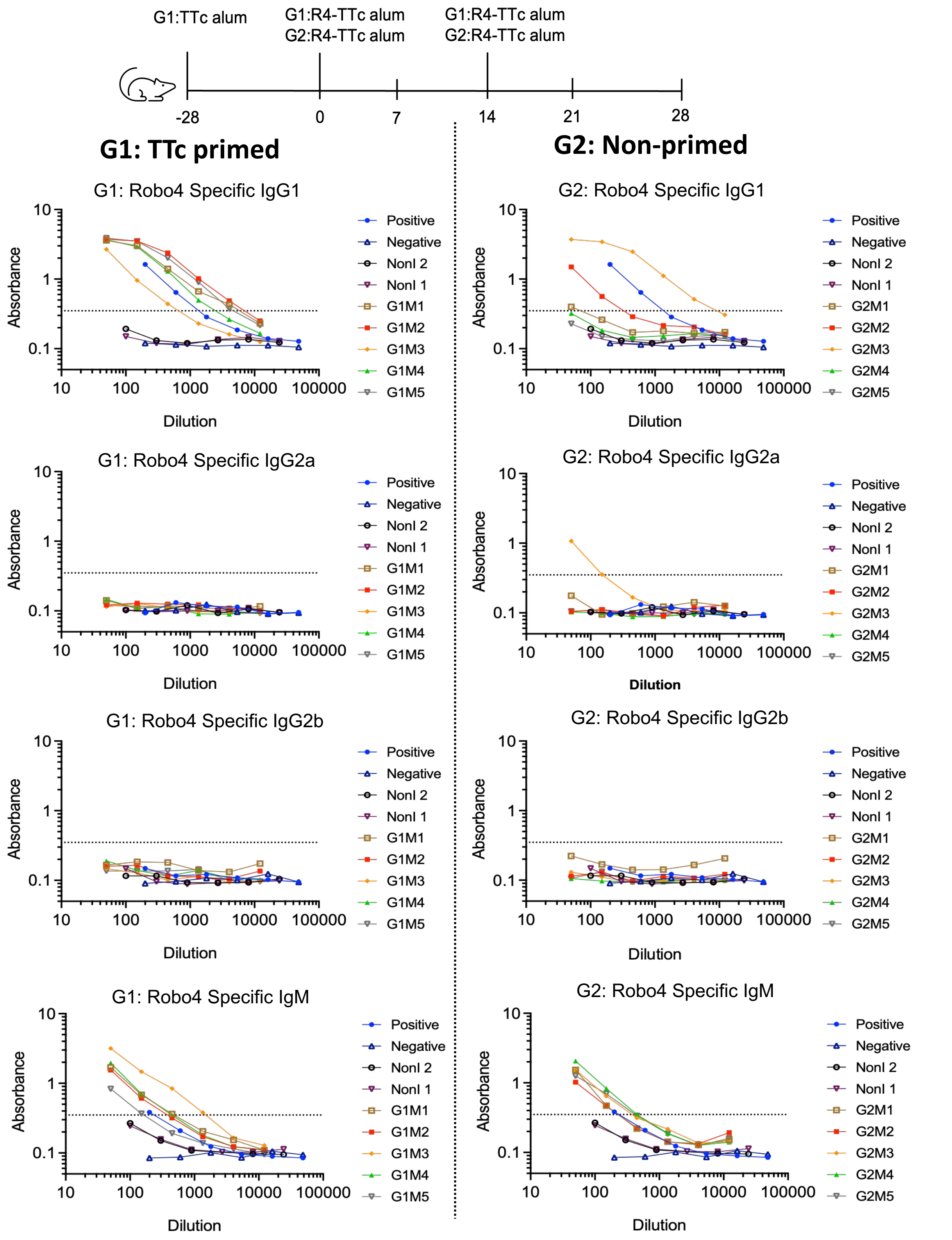

Suppl Figure S4. The induction of Robo4 specific antibody after injection of Robo4-TTc Alum

Group1 (G1) are C57BL6 mice primed with TTc in alum. Group2 (G2) are non-primed C57BL6 mice. 4 weeks later, G1 and G2 were injected with R4-TTc in alum, 2 weeks later, both groups were boost again. Robo4 specific IgG1, IgG2a, IgG2b and IgM were measured in serum at day28 after the second boost with R4-TTc in alum.

Left: Samples are from Group1(G1), TTc-Primed.

Right: Samples are from Group2 (G2), non-primed.

Each line represents an individual animal. The dashed indicates the detection limit.

NonI: Non-immunised. G1M1: Group1 Mouse1, G1M2: Group1 Mouse2..etc.

G2M1: Group2 Mouse1, G1M2: Group2 Mouse2..etc

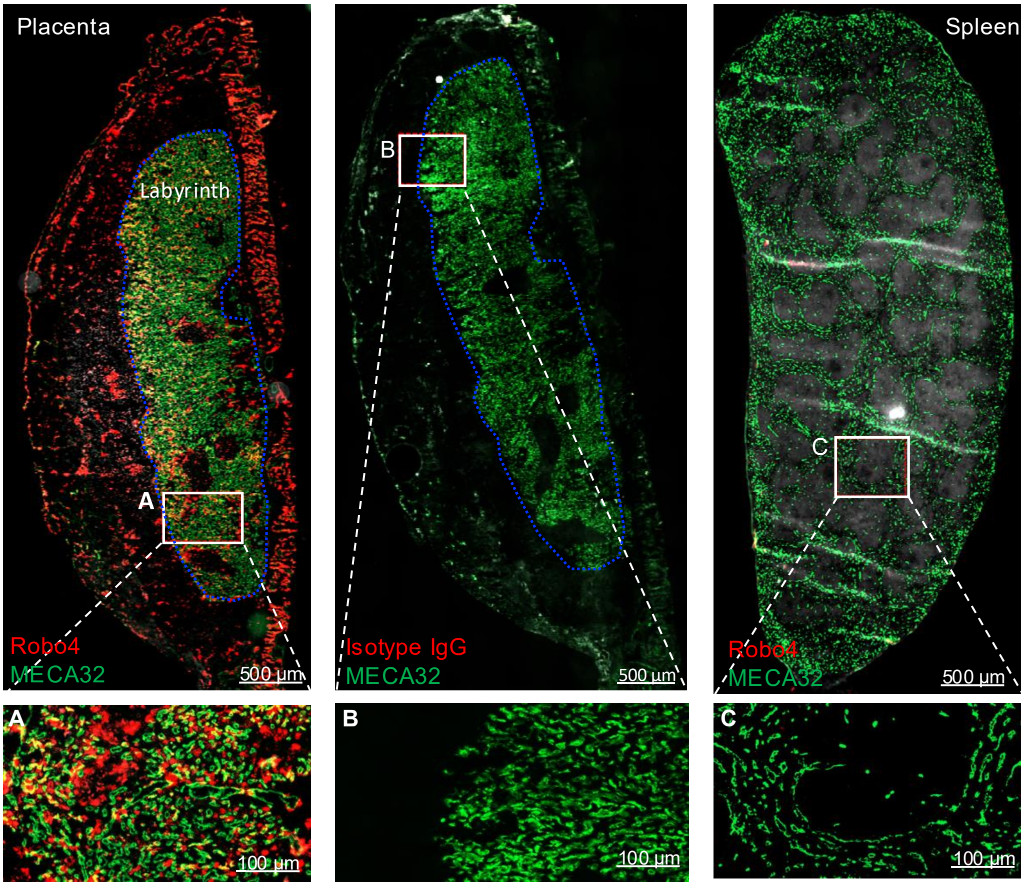

Suppl Figure S5. Immunofluorescent staining to identify Robo4 expression. Immunofluorescent staining Robo4 and pan-endothelial cell antigen (MECA32) on one mature mouse placental section. Areas marked by the blue dotted lines indicate the placental labyrinth. **Left (A):** Expression of Robo4 and MECA32 on the labyrinth. **Middle (B):** Expression of MECA32 and Rabbit Isotype IgG on the same placenta section. **Right(C):** Robo4 and MECA32 expression on a healthy mouse tissue (spleen).

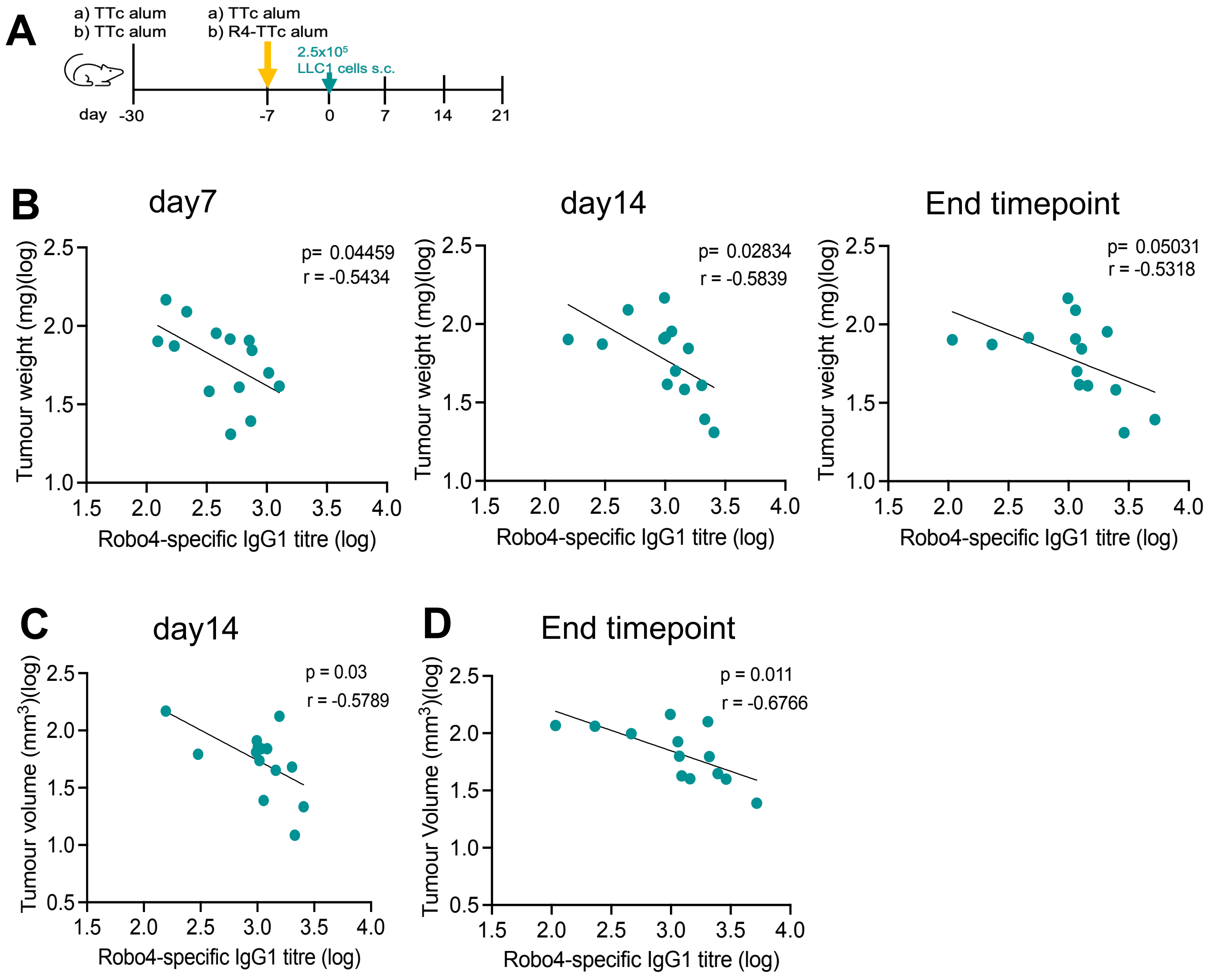

Suppl Figure S6. Correlation between the Robo4-specific IgG1 and the tumour weight and tumour volume.

A: Experiment protocol. Mice were primed with TTc in alum, 3 weeks later boosted with R4-TTc alum or TTc alum. LLC1 cells were injected s.c in the right flank of the mouse at d7 after the boost. Antibody was assessed from serum harvested at different timepoint after the tumour cell transplantation.

B) Correlation of Robo4 specific IgG1 at d7, d14, and d21 with final tumour weight

C) Correlation of Robo4 specific IgG1 titres with tumour volume at d14

D) Correlation of Robo4 specific IgG1 titres with tumour volume at the end timepoint

Each symbol represents one mouse. Data were combined from two independent experiments. Two-tailed Compute Pearson correlation coefficients for samples from R4-TTc treatment group.

Day7: some tumours were very small, difficult to measure, so tumour volume was not calculated. There is no correlation between Robo4 specific IgG1 titres and volume for this timepoint.

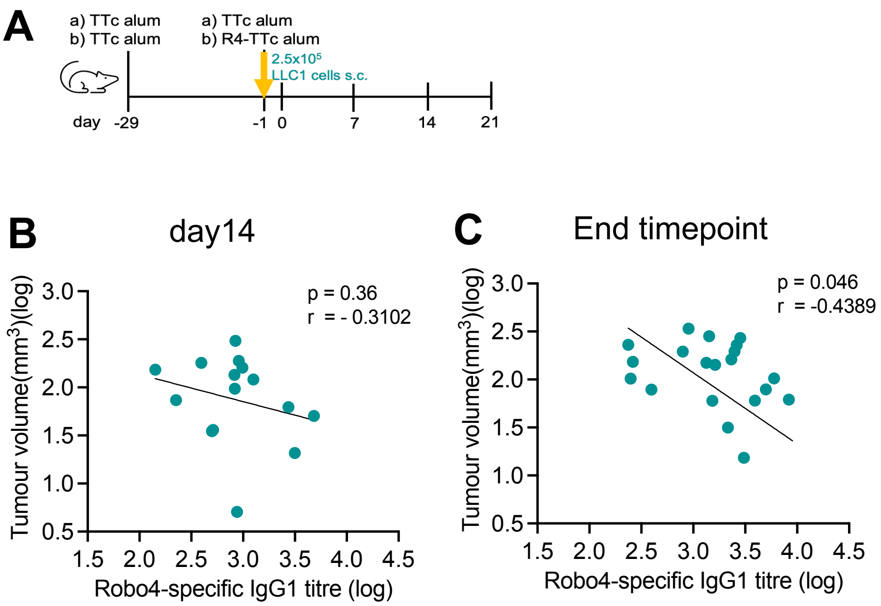

Supp Figure S7. Correlation between the Robo4-specific IgG1 and tumour volume.

A) Experiment protocol: Mice were primed with TTc in alum, 4 weeks later boost with R4-TTc in alum or TTc in alum. LLC1 cells were injected s.c in the right flank of the mouse at one day after the boost.

B) Correlation of Robo4 specific IgG1 titres with tumour volume at d14

C) Correlation of Robo4 specific IgG1 titres with tumour volume at the end timepoint.

Each symbol represents one mouse. Data were combined from three independent experiments. Two-tailed Compute Pearson correlation coefficients for samples from R4-TTc treatment group.

Day7: some tumours were very small, difficult to measure, so tumour volume was not calculated. There is no correlation between Robo4 specific IgG1 titres and volume for this timepoint.

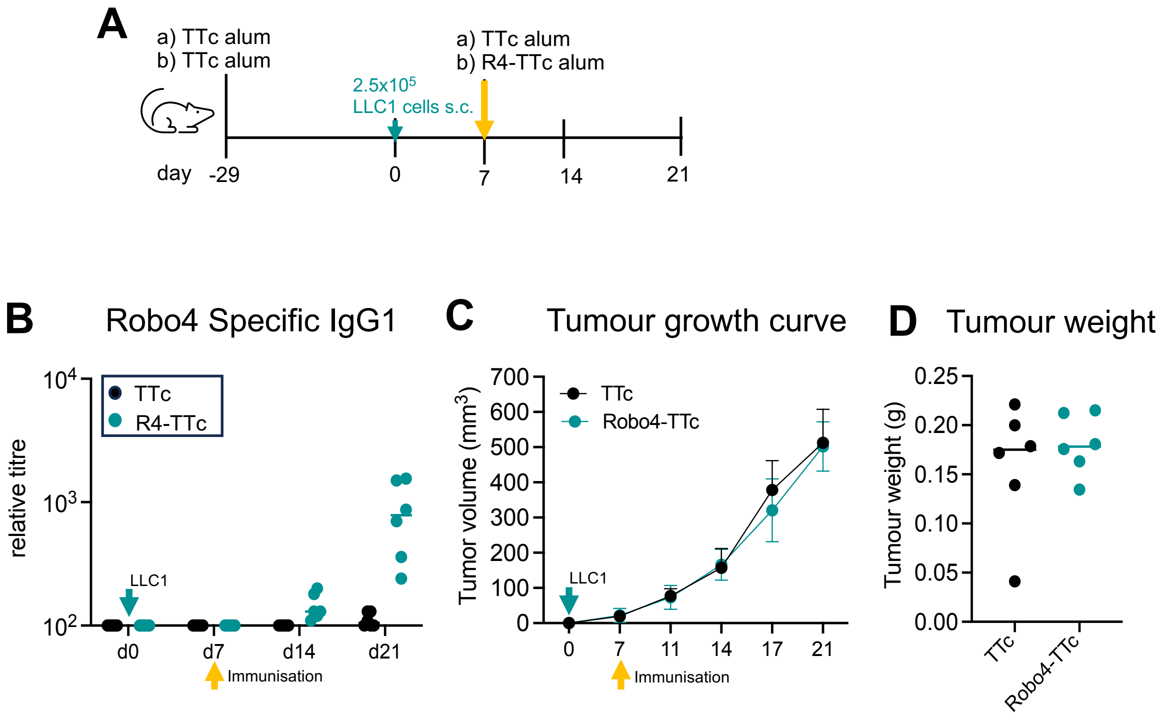

Suppl Figure S8. Tumour size if tumour cell transplantation before Robo4-TTc vaccination

A)Experiment protocol: Mice were primed with TTc in alum. After 4 weeks (d0) LLC1 cells were injected s.c in the right flank of the mouse, 7 days later mice were boosted with R4-TTc in alum or TTc in alum.

B) Robo4-specific IgG1 antibody detected in the sera by ELISA.

C) Tumour growth curve, plotted as mean± SD.

D)Tumour weight at d21 after Robo4-TTc vaccination

Each symbol represents one mouse.

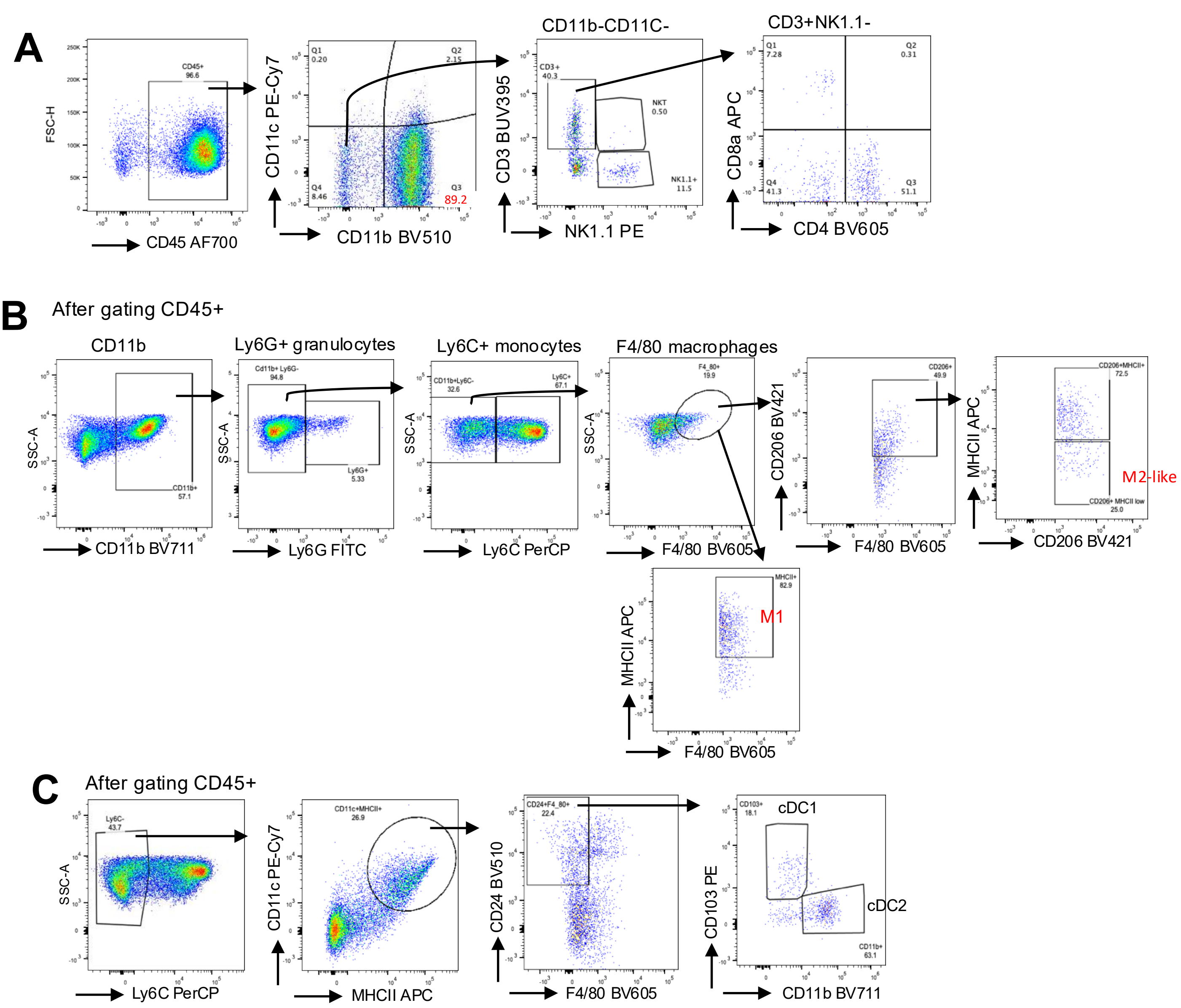

Suppl Figure S9. Flow cytometric gating to identify immune cells in tumours.
Experiment protocol is shown in Figure 4E.
Mice were primed with TTc in alum, 4 weeks later boost with R4-TTc in alum or TTc in alum. LLC1 cells were injected s.c in the right flank of the mouse at one day after the boost.
A) Representative flow cytometric gating to identify T cells from dispersed tumour tissue (CD11b$^-$CD11c$^-$ CD3$^+$) from live CD45+cells.
B) Representative flow cytometric gating to analyze CD11b$^+$ cells, further to identify CD11b$^+$Ly6G$^+$ granulocytes, CD11b$^+$Ly6G$^-$Ly6C$^+$ monocytes, and CD11b$^+$Ly6G$^-$Ly6C$^-$ F4/80$^+$ macrophages, which further to gate M2-Like cells: CD206$^+$MHCII$^-$
C) Representative flow cytometric gating to identify Ly6C$^-$CD11c$^+$MHCII$^+$F4/80$^-$ dendritic cells.

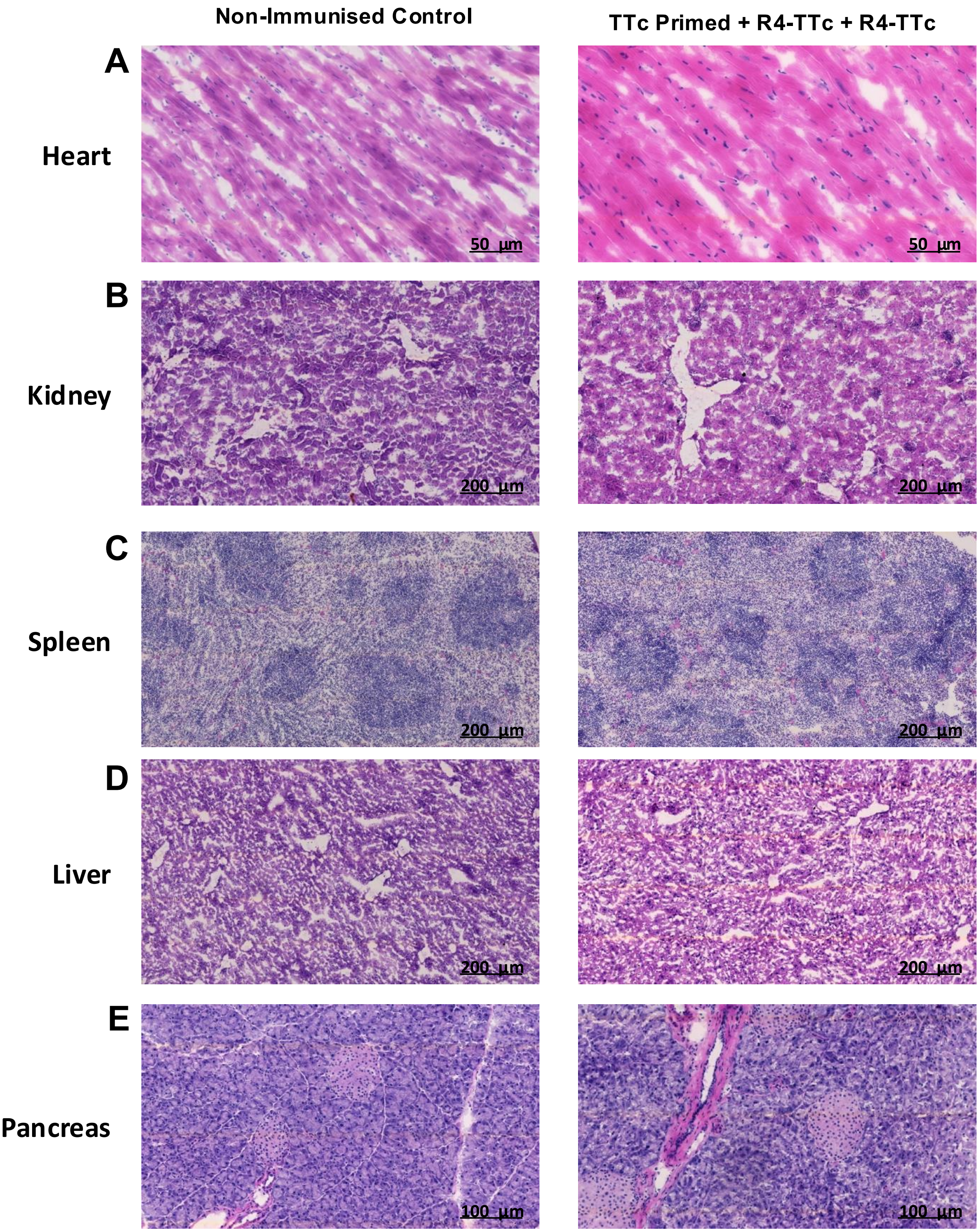

Suppl Figure S10. Haematoxylin and eosin staining of tissues from non-immunised and Robo4 vaccinated mice.
Tissues including heart, kidney, spleen, liver and pancreas were harvested at the endpoint of the experimental protocol outlined in Figure 2D. The slides were stained with H&E staining. Representative images of **A)** Heart, **B)** Kidney, **C)** Spleen, **D)** Liver and **E)** Pancreas from 4 mice per experimental group.

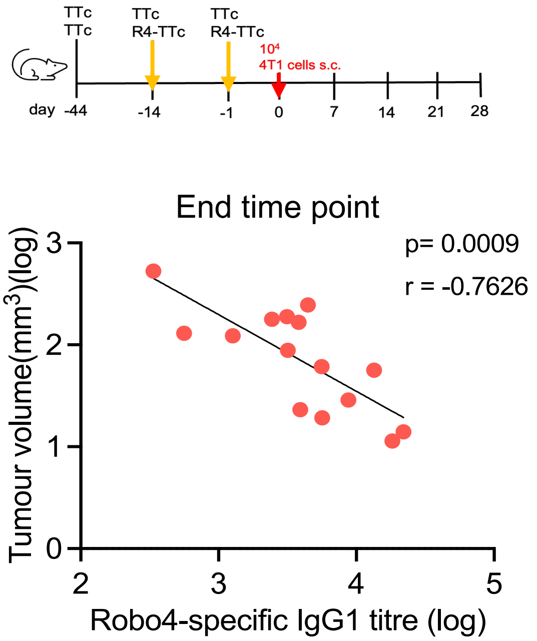

Suppl Figure S11. Correlation between the Robo4-specific IgG1 and the final tumour volume.

Experiment protocol. Mice were primed with TTc in alum, 4 weeks later boost with R4-TTc in alum or TTc in alum. 4T1 cells were s.c. injected into 4th mammary fat pad one day after the second boost with R4-TTc in alum or TTc in alum.

Correlation of Robo4 specific IgG1 titres with tumour volume at the end timepoint. Each symbol represents one mouse. Data were combined from three independent experiments. Two-tailed Compute Pearson correlation coefficients for samples from R4-TTc treatment group.

Day7, day14: some tumours from the R4-TTc group were very small and difficult to measure, so tumour volume was not calculated and there is no correlation between Robo4 specific IgG1 titres and tumour volume for these timepoints.
