# Supplementary material for: Vaccination against tumour endothelial marker Robo4 inhibits tumour growth
Source: Immunother Adv. 2026 Jun 25;6(1):ltag005. doi: 10.1093/immadv/ltag005 (PMC13296997; doi:10.1093/immadv/ltag005)
Supplement: ltag005_Supplementary_Data [file ltag005_supplementary_data.zip › SupplementaryMatherialsandMethods.pdf]

Supplementary Materials and Methods for

Vaccination against tumour endothelial marker Robo4 inhibits tumour growth

Fernanda Escobar-Riquelme[1], Muhammet Ali Kara[1], Michael J Price[1], Angela Hidalgo-Gajardo[1 2], Hayley L Carr [3], David Bending[1], Roy Bicknell[4], Natalia Savelyeva[5], Kai-Michael Toellner[1 6 *], Yang Zhang[1 6 *]

Affiliations

[1] Department of Immunology and Immunotherapy, School of Infection, Inflammation and Immunology, College of Medicine and Health, University of Birmingham, UK
[2] Departamento de Fisiopatología, Facultad de Ciencias Biológicas, Universidad de Concepción, Chile
[3] Bioinformatics, The Babraham Institute, Babraham Research Campus, Cambridge, UK
[4] Department of Cardiovascular Sciences, College of Medicine and Health, The University of Birmingham, UK
[5] Molecular and Clinical Cancer Medicine, Institute of Systems, Molecular and Integrative Biology, Faculty of Health and Life Science, University of Liverpool, UK
[6] Immunology Program, The Babraham Institute, Babraham Research Campus, Cambridge, UK
[*] Shared Corresponding authors

Main correspondence to
yang.zhang@babraham.ac.uk

The PDF file includes
Materials and Methods
Suppl Figure S1 – S11
Suppl Table 1 – 11

Supplementary Materials and Methods

Cell culture

HEK293 cells (ATCC; CRL-1573) were grown in Minimum Essential Medium (MEM) (Sigma; M2279), supplemented with 10% v/v Fetal Bovine Serum Heat Inactivated (FBS-HI) (Sigma; F9665); 2 mM L-glutamine (Gibco; 25030081); 1 mM sodium pyruvate (S8636-100ML); and 100 U/mL penicillin and 100 µg/mL streptomycin (Gibco; 15140-122), referred to as MEM complete medium. During the selection of stably transfected cells, the MEM complete medium was supplemented with 500 µg/mL G418 disulfate salt (Sigma; A1720). After selection, the medium was supplemented with 300 µg/mL G418 disulfate salt.

Generation of the vectors for mammalian expression of murine Robo4 protein, TTc carrier and the genetically linked Robo4-TTc protein.

The sequence of the extracellular region of mouse Robo4 (R4) was obtained from Roy Bicknell. The sequence of TTc was obtained from Dr. Natalia Savelyeva. The Fc-tagged R4 and TTc sequences were codon-optimised for mammalian expression and designed to be flanked by the restriction enzymes NheI and ApaI (Integrated DNA Technologies, IDT). Linker sequences were added between R4 or TTc and the Fc-tag. Sequences were received in the standard pUCIDT plasmid. The R4-TTc expression plasmid was designed and built by assembling the two previous sequences and keeping the Fc-tag from the TTc insert. R4 and TTc DNA sequences are shown in Suppl Table 2 and 3.

Briefly, for the construction of these vectors, the mammalian expression vector pcDNA3.1-mGFP was used as the backbone vector for subcloning. The membrane-bound green fluorescent protein (mGFP) sequence was excised by restriction enzyme digestion (ApaI and NheI), and the backbone vector was purified using the QIAquick Gel Extraction Kit (Qiagen; 28704). The Fc-tagged R4 and TTc sequences were excised using the same restriction

enzymes, gel purified, and subcloned separately into the multiple cloning sites of the pcDNA3.1 vector. All plasmids are listed in Suppl Table1.

To construct a vector containing genetically linked R4 and TTc, a DNA assembly was performed using the pcDNA3.1-R4 and pcDNA3.1-TTc vectors previously generated. After the DNA assembly, the full construct consisted of R4 + Linker 1 + TTc + Linker 2 + Fc-tag (Suppl. Figure S2). The full DNA sequence can be found in Suppl Table 4. Competent bacteria were transformed with the assembled product. Colonies were assessed by Colony PCR with primers targeting the junction regions and both linkers. The pcDNA3.1 R4-TTc assembly was analysed by restriction enzyme digestion and sequencing (Suppl. Figure S2).

Protein production

HEK293 cells were transfected with the different vectors and protein expression was assessed by SDS-PAGE and western blot. Polyethyleneimine (PEI) (Sigma; 408727) was used as a transfection reagent. Stably transfected cell lines were generated and selected with an optimised dose of G418 disulfate salt. The presence of proteins in the supernatants was confirmed by SDS-PAGE and western blot. The R4-TTc protein was detected with anti-TTc and anti-R4 antibodies, showing overlapping signals of at the same size. The protein TTc, R4, and R4-TTc sequences are shown on Suppl. Table 5-7, and theoretical protein sizes are shown on Suppl. Table 8.

Protein purification

Purifications were guided by Dr. Margaret Goodall. Supernatant was 0.22µm filtered and passed through a HiTrap 1 ml Protein A HP column (GE Healthcare Cytiva; 17040203). The protein was eluted with 0.1 M citric acid (Melford; C3450), pH 2.8, and immediately dialysed

against PBS on a Slide-A-Lyze Dialysis Cassette (Thermo Scientific; 66455) at 4°C. Purification was confirmed by SDS-PAGE and western blot (Suppl Figure S3).

Protein detection by SDS-PAGE and Western blot

For protein electrophoresis and western blot, equal amounts of protein were prepared in NuPAGE LDS sample buffer (Invitrogen; NP0007) with 50 mM final concentration of NuPAGE Sample Reducing Agent (Invitrogen; NP0004) and incubated at 70°C for 10 min. Samples were resolved in a NuPAGE 4-12% gradient Bis-Tris precast-gel (Invitrogen; NP0321). Gels were run in NuPAGE MOPS SDS Running Buffer (Novex; NP0001) with 0.25% v/v of NuPAGE Antioxidant (Invitrogen; NP0005) for 45min. Then gels were then stained with Coomassie Blue stain Simply Blue Safe stain (Invitrogen; LC6060) for 1 h. Gels were washed with dH_2O before image acquisition in a ChemiDoc Imaging System (Bio-Rad).

For immunoblotting, a blocking buffer was prepared with 5% w/v Marvel skimmed milk in Tris-buffered saline (TBS). TBS was comprised of 150 mM Tris-HCl (Sigma; T3253), 45 mM Tris-base (Sigma; T1503) and 1.5 M NaCl (Melford Biolaboratories, S0520). The wash buffer consisted of PBS 0.1% v/v Tween-20. Protein samples were transferred from a gel to 0.45 µm PVDF transfer membrane (Thermo Scientific; 88519) by wet transfer in NuPAGE Transfer Buffer (Novex; NP0006) for 2 h. After incubation in blocking buffer for 1 h at room temperature (RT), membranes were stained with primary antibodies at 4°C for 16 h. Membranes were washed and then incubated with secondary antibodies for 1h at RT. Finally, gels and membranes were scanned on the ChemiDoc Image System and edited using the Image Lab Standard Edition v6.0.1 software (Bio-Rad). Antibodies are listed in Suppl Table10 and 11.
